# hnRNPUL1 has a dead polynucleotide kinase domain that regulates RNA and protein interactions

**DOI:** 10.1016/j.isci.2026.115360

**Published:** 2026-03-14

**Authors:** Carmen V. Apostol, Ang Li, Peter Daniels, Llywelyn Griffith, Johnathan Cooper-Knock, Elisa Aguilar-Martinez, Ivaylo D. Yonchev, Ashleigh G.R. Whelan, Pamela J. Shaw, Ian M. Sudbery, Stuart A. Wilson

**Affiliations:** 1 Sheffield Institute for Nucleic Acids, School of Biosciences, The University of Sheffield, Firth Court, Western Bank, Sheffield S10 2TN, UK; 2 Sheffield Institute for Translational Neuroscience, The University of Sheffield, Sheffield S10 2HQ, UK; 3 NIHR Sheffield Biomedical Research Centre, Sheffield Teaching Hospitals NHS Foundation Trust, Sheffield S10 2JF, UK

**Keywords:** Molecular interaction, Protein structure aspects

## Abstract

hnRNPUL1 is a nuclear RNA-binding protein involved in both pre-mRNA splicing and DNA double-strand break repair. Using AlphaFold, we show that hnRNPUL1 has a central folded region consisting of tightly juxtaposed SPRY and dead polynucleotide kinase (dPNK) domains flanked by intrinsically disordered regions (IDRs). The dPNK domain binds both nucleotides and RNA. Remarkably, polynucleotide kinase activity can be reactivated with a single amino acid substitution. Mutations altering nucleotide binding also change the ability of the entire protein to bind RNA and regulate homotypic versus heterotypic protein interactions driven by the IDRs. A mutation that prevents nucleotide binding also destabilizes the protein. In a small number of amyotrophic lateral sclerosis patients, we identify rare coding variants in the *HNRNPUL1* gene, which alter the ability of hnRNPUL1 to bind nucleotides, RNAs, and FUS. Together, these data establish that hnRNPUL1 utilizes its dPNK domain to regulate interactions with itself, RNA, and other proteins.

## Highlights

• hnRNPUL1 SPRY-dPNK domain binds NTPs and uncapped RNAs via a ligand channel
• N456D point mutation rescues the polynucleotide kinase catalytic activity
• SPRY-dPNK domain interacts directly with the disordered C-terminal domain
• SPRY-dPNK domain regulates homotypic versus heterotypic interactions with FUS

## Graphical abstract

**Figure:**
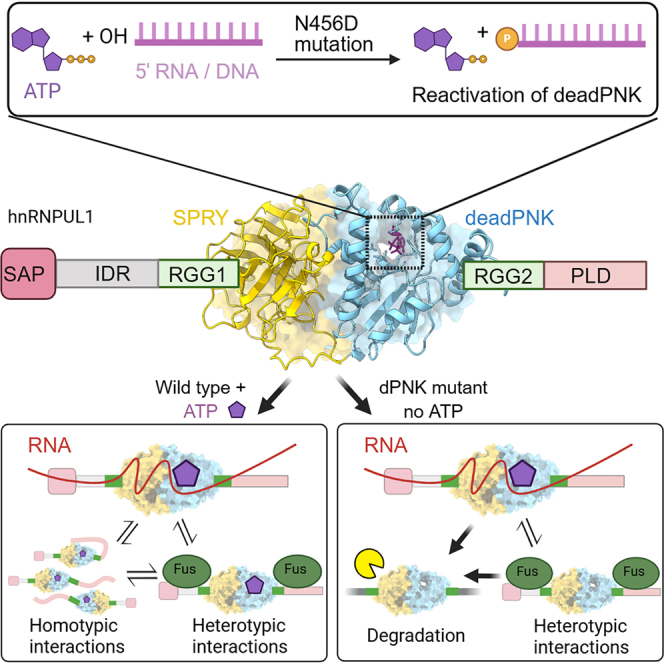

## Introduction

The heterogeneous nuclear ribonucleoproteins (hnRNPs) bind heterogeneous nuclear RNA and are largely composed of pre-mRNA species.^1^ They play roles in transcription, RNA processing, stability, localization, and translation.^2^ hnRNPUL1 is one of the largest hnRNPs, sharing sequence homology with hnRNPU and hnRNPUL2. It was originally identified as a protein whose overexpression overcame the mRNA export block imposed by adenovirus infection of cells^3^ and was subsequently found to associate with the mRNA export factor Nxf1.^4^ However, its role in cellular mRNA export is currently unclear. hnRNPUL1 is also involved in DNA double-strand break (DSB) repair, where it is recruited to sites of damage via NBS1. As part of the MRE11-RAD50-NBS1 (MRN) complex, NBS1 promotes RNA polymerase II (RNA Pol II) transcription at DSBs, leading to the production of dilncRNAs.^5^ Further, hnRNPUL1 has been shown to work together with hnRNPUL2 to drive DNA end resection, ATR signaling, and recruitment of BLM helicase, though its molecular role in these processes remains unclear.^6^ hnRNPUL1 is involved in pre-mRNA splicing, specifically alternative splicing, though the molecular mechanisms are again not understood.^7^^,^^8^ It is further implicated in the repression of histone gene transcription in cell cycle-arrested cells through an interaction with the U7 snRNP,^9^ and a large-scale analysis of chromatin binding identified hnRNPUL1 as the most highly enriched nuclear RNA-binding protein on snRNA genes, indicating a potential role in their biogenesis.^10^

Sequence variants in hnRNP genes lead to multiple diseases, including frontotemporal dementia and neurodevelopmental disorders.^11^^,^^12^^,^^13^ Several hnRNPs are implicated in amyotrophic lateral sclerosis (ALS), including FUS, hnRNPA2B1, and hnRNPA1. A hallmark of these proteins is a prion-like domain that can self-associate and phase separate.^14^ Interestingly, hnRNPUL1 has a C-terminal prion-like domain with similar biochemical properties to FUS and is predicted to phase separate at physiological concentrations.^14^ Moreover, hnRNPUL1 binds the ALS-causative FET family of proteins (FUS, EWSR1, and TAF15).^15^^,^^16^ It is also sequestered by C9orf72 repeat expansion RNA, which is commonly associated with ALS,^17^ and, recently, an hnRNPUL1 variant (p.P54Q)^18^ was identified among a cohort of ALS patients. Together, these data suggest that hnRNPUL1 may play a role in ALS. In addition, hnRNPUL1 is implicated in B cell precursor acute lymphoblastic leukemia (ALL), where a chromosomal translocation fuses the hnRNPUL1 CTD to the DNA-binding domain of MEF2D to generate an aberrant transcription factor.^19^^,^^20^^,^^21^ Finally, hnRNPUL1 was identified as a candidate gene in two siblings with congenital limb malformations.^7^

Despite a number of functional studies implicating hnRNPUL1 in diverse processes such as mRNA export, DNA repair, and transcription, there is surprisingly little structural or biochemical information describing this protein. Inspection of the Uniprot entry for hnRNPUL1 (Q9BUJ2) reveals an N-terminal annotated SAP domain (amino acids [aa] 3–37) for which there is an NMR structure (PDB: 1ZRJ) and an SPRY domain (aa 191–388). Furthermore, the original work describing hnRNPUL1^3^ identified a central nucleotide-binding region, adjacent to the more recently annotated SPRY domain, with characteristic Walker A and B motifs,^22^ hereafter abbreviated as WA and WB, respectively. This study^3^ also identified RGG boxes associated with RNA binding, and these sites are subject to arginine methylation by HRMT1L2.^23^ The ENCODE project utilized eCLIP to analyze the RNA-binding activity of hnRNPUL1 *in vivo*, which revealed extensive binding to intronic RNA and other non-coding RNA species,^24^ consistent with its reported role in alternative splicing.^7^^,^^8^ However, it remains uncertain whether hnRNPUL1 binds to specific sequence motifs or structures within RNA *in vivo*.

Here, we used a mixture of AlphaFold modeling and biochemical assays to characterize hnRNPUL1 and examine the impact of coding variants on its biological activity. We identify a single globular central folded domain, which regulates the RNA- and protein-binding activity of adjacent intrinsically disordered regions (IDRs).

## Results

### The core of hnRNPUL1 shares structural homology with mammalian polynucleotide kinase phosphatase

**Figure 1:**
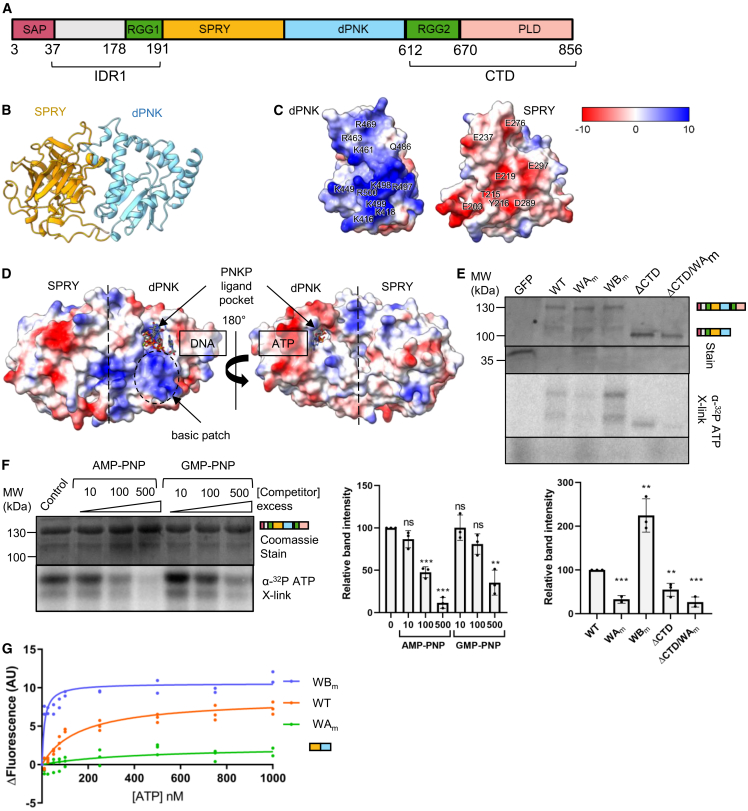
hnRNPUL1 has a central folded domain that binds to nucleotides (A) Domain model of hnRNPUL1. (B) AlphaFold3 model of the predicted central folded domain of hnRNPUL1 (200–601). The SPRY region (200–417) is shown in gold, and the dPNK region is shown in light blue. (C) Surface charge representation of the interface between the isolated hnRNPUL1 SPRY and dPNK regions. Surface potential calculated and displayed using “Coulombic” command in ChimeraX, expressed as kcal/(mol·e) at 298 K. (D) Surface charge representation of the central folded domain of hnRNPUL1(415–600), overlaid onto PNKP (307–522) in complex with DNA and ATP (PDB: 3ZVN), with an RMSD for aligned Cα pairs of 0.971 Å. A basic patch leading into the binding pocket is encircled. (E) ATP-binding assay using α-^32^P ATP UV crosslinked to different forms of hnRNPUL1. Inset: Quantification of band intensities relative to WT, with *p* values calculated using the unpaired *t* test (*n* = 3). Data are represented as the mean ± SEM. (F) Nucleotide competition of full-length hnRNPUL1 UV crosslinked to α-^32^P ATP and varying amounts of non-hydrolysable ATP or GTP analogues. Inset: Quantification of band intensities relative to an untreated control, with *p* values calculated using the unpaired *t* test (*n* = 3). Data are represented as the mean ± SEM. (G) Tryptophan fluorescence quenching of WT or mutant SPRY-dPNK truncations upon titration of ATP. Data are represented as the mean ± SEM. Statistical significance is shown by ∗∗, indicating *p* < 0.01, and ∗∗∗, indicating *p* < 0.001. See also Figure S1.

A schematic of the domains of hnRNPUL1 is presented in Figure 1A. Based on biochemical results discussed later, we named the central nucleotide triphosphate (NTP)-binding region, dPNK, for dead polynucleotide kinase. Protein sequence analysis using IUPred3^25^ confirmed the presence of an ordered central region flanked by disordered N- and C-terminal arms, termed IDR1, and a carboxy-terminal domain (CTD). Both IDR1 and the CTD harbor RG/RGG peptides associated with RNA-binding activity, labeled RGG1 and RGG2^26^ (Figures 1A and S1A).

In the absence of experimentally derived structural information, we used AlphaFold v3.0^27^ to predict structures of the central folded domains of hnRNPUL1 (Figure 1B). The model indicates that the SPRY and dPNK regions of hnRNPUL1 come together in the tertiary structure to form a single globular domain, joined by electrostatic interactions, as well as hydrophobic contacts, at their interface (Figure 1C). The SPRY-dPNK region was modeled with an average pLDDT value of 91.1, with small predicted aligned errors between 5 and 10 Å, indicating a high degree of confidence in the quality of prediction (Figures S1B and S1C).

To characterize the hnRNPUL1 folded core, we searched for structural homologues with known functions, using PDBeFold^28^ and Phyre2.^29^ We failed to detect homologues to the entire SPRY-dPNK core; however, Phyre2 aligned the hnRNPUL1 dPNK region to the kinase domain of mammalian polynucleotide kinase phosphatase (PNKP), with >90% confidence (Figure S1D). The AlphaFold model was superimposable onto the apo-PNKP structure (PDB: 3ZVL) with a root-mean-square deviation (RMSD) of 0.8 Å across 284 Cα pairs. The dPNK domain displays a large surface basic patch lining the entrance into a pocket, which corresponds to the ligand-binding pocket of the PNKP kinase (Figure 1D). The polynucleotide kinase domain of PNKP binds two types of ligands, NTPs and nucleic acids with -OH 5′ ends; hence, we assessed hnRNPUL1’s ability to bind both.

### hnRNPUL1 binds NTPs

We tested NTP binding to hnRNPUL1, using UV crosslinking of α-^32^P ATP to mutant or truncated forms of the protein. We used FLAG-tagged proteins directly purified from human cells for these experiments because preliminary assays using proteins purified from *E. coli* yielded poor biological activity. For clarity, we use the term “mutants” to describe engineered changes in the coding sequence with resulting changes in the amino acid sequence, whereas we use the term “variant” to describe protein sequence changes identified through sequencing of individuals. We tested mutants with a disrupted WA motif (G433A, K434A, and T435A), hereafter referred to as WA_m_, and a disrupted Walker B motif (D505A), hereafter referred to as WB_m_ (Figure S1E). hnRNPUL1 formed a discrete protein-ATP complex, even in the absence of its positively charged arginine-rich CTD, implying a direct interaction via the SPRY-dPNK domain (Figure 1E). The WA_m_ mutation reduced ATP binding, while WB_m_ enhanced it. The D505 residue mutated in WB_m_ is predicted to hydrogen bond with both the WA residue T435 and the water molecules around the ATP pocket (Figure S1E). Disrupting this interaction network may lead to a local conformational change, either driving the enhanced ATP binding or preventing its release. We tested hnRNPUL1’s NTP preference with a competition assay by using non-hydrolysable analogs of both ATP and GTP (Figure 1F). Both competitors displaced the α-^32^P-ATP in a concentration-dependent manner, although the ATP analog emerged as a stronger competitor. This finding is consistent with the ability of bacteriophage T4 PNK to use a range of NTPs as phosphate donors.^30^

To estimate the binding affinity for ATP of the dPNK mutants, we monitored the intrinsic tryptophan fluorescence change of the SPRY-dPNK domains upon titration of ATP. Binding constants derived for wild-type (WT) and WB_m_ proteins supported the UV crosslinking results: K_d(WT)_ = 164 ± 22 nM and K_d(WBm)_ = 19 ± 8.5 nM, with WA_m_ showing negligible binding (Figure 1G). Two tryptophan residues, W477 and W437, are located close to the ATP-binding site and likely contribute to the fluorescent signal quenching through conformational changes in their local microenvironment (Figure S1F). Moreover, W477 is located in a region predicted with low confidence (<70%) in the AlphaFold model (Figure S1C), which may arise from local flexibility of this region due to a ligand-induced conformational change. Such a motion can be modeled on the known apo- and ligand-bound structures of PNKP, where ATP binding elicits closing of the structure around the binding pocket^31^ (Figure S1F).

### hnRNPUL1 harbors a novel RNA-binding domain in the hnRNP family

**Figure 2:**
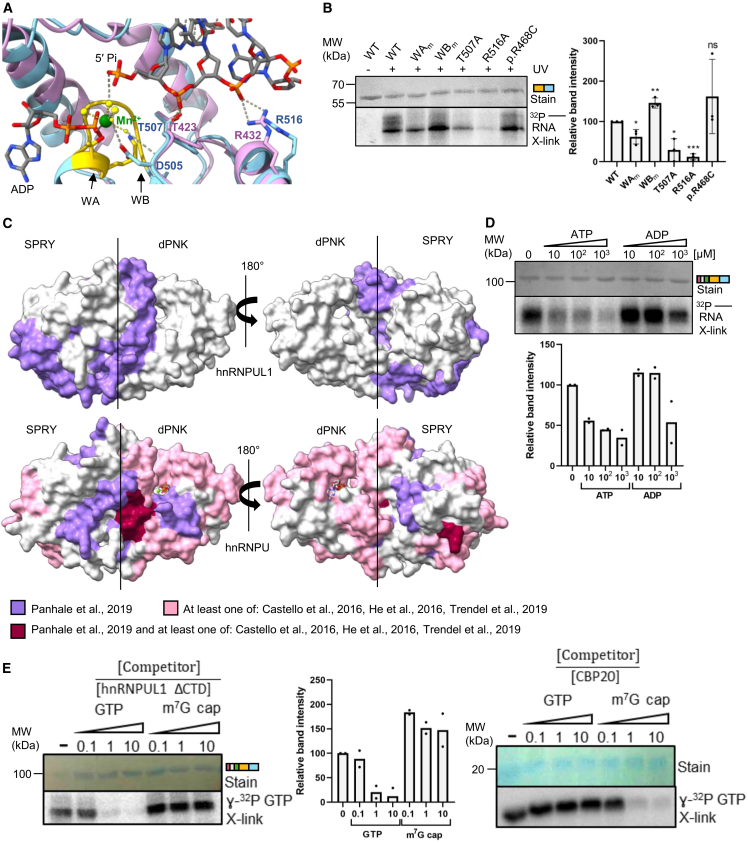
The SPRY-dPNK domain binds RNA (A) Structural comparison between the ligand pockets of the hnRNPUL1 AlphaFold3 model (blue) and PNKP (violet, PDB: 3ZVN). Residues of the WA (gold) and WB motifs are shown in stick form, along with the conserved residues T507 and R516. Dashed lines denote hydrogen bonds. (B) UV crosslinking of WT or mutant SPRY-dPNK to 5′ ^32^P-labelled RNA oligonucleotide. Inset: Quantification of band intensities relative to WT, with *p* values calculated using the unpaired *t* test (*n* = 3). Data are represented as the mean ± SEM. (C) AlphaFold3 predictions of the hnRNPUL1 (191–612, top) and hnRNPU (268–686, bottom) SPRY-dPNK cores highlighting RNA-interacting peptides identified in various global mass spectrometry studies. More details in Figure S2. (D) UV crosslinking of hnRNPUL1 ΔCTD to a 5′ ^32^P-labelled RNA oligonucleotide and varying amounts of ATP or ADP. Inset: Quantification of band intensities for two replicates, with the bar showing the mean value. (E) Competition assay with hnRNPUL1 ΔCTD (left) or CBP20 as positive control (right) UV crosslinked to γ-^32^P GTP and varying amounts of unlabeled GTP or m^7^G cap analogue. Inset: Quantification of band intensities for two replicates of the experiment in the left relative to an untreated control with the bar showing the mean value. See also Figure S2.

The structural homology between dPNK and PNKP raises the possibility that hnRNPUL1 could bind nucleic acids via the central ligand pocket independently of the RGG-containing CTD and SAP domains. Superimposition of the AlphaFold model onto the ligand-bound PNKP structure (PDB: 3ZVN) revealed two structurally conserved residues located within hnRNPUL1’s basic patch, T507 and R516 (Figure 2A). The equivalent T423 and R432 in PNKP are involved in stabilizing and positioning the phosphate backbone of a single-stranded DNA oligonucleotide 5′ end bound to PNKP’s kinase domain.^31^

We tested the nucleic acid-binding activity of hnRNPUL1 with UV crosslinking of the SPRY-dPNK truncation to a ^32^P end-labelled RNA oligonucleotide in the absence of NTPs (Figure 2B). Because hnRNPUL1 has been previously shown to bind RNA homopolymers,^3^ and eCLIP^24^ revealed binding across a wide range of RNA substrates, we chose a non-specific RNA sequence for these studies, as detailed in STAR Methods. The SPRY-dPNK_WT_ domain bound the RNA oligonucleotide, while WA_m_ and WB_m_ displayed reduced and enhanced binding, respectively. Because these assays were carried out in the absence of NTPs, the different binding activities associated with WA_m_ and WB_m_ mutants may arise from subtle disruption of the dPNK fold at its core, which is expected as the residues involved are predicted to form a hydrogen-bonding network with ATP and a metal ion (Figure S1E). Mutation of the two conserved basic patch residues T507A and R516A significantly decreased RNA binding to the SPRY-dPNK domain, indicating conservation of nucleic acid-binding sites between hnRNPUL1 and PN6KP.

Further supporting the SPRY-dPNK cores of hnRNPUL1 and the related protein hnRNPU as RNA-binding domains, peptides from these regions have been recently identified in global mass spectrometry studies of RNA-binding proteins^32^^,^^33^^,^^34^^,^^35^ (Figure 2C). More RNA-crosslinked peptides were mapped to hnRNPU’s core compared to hnRNPUL1, which may reflect the intracellular abundance of each protein (∼2 × 10^6^ hnRNPU copies and 1.8 × 10^5^ hnRNPUL1 copies per HeLa cell^36^). Additional RNA-binding peptides were mapped in hnRNPU family of proteins to the SAP, IDR1, and the well-characterized RGG RNA-binding domains (Figure S2), indicating that this protein family contacts RNA through multiple domains *in vivo*.

To explore the interplay between ATP and RNA binding to hnRNPUL1’s SPRY-dPNK pocket, we assessed binding to 5′-monophosphorylated RNA in the presence of ADP or ATP (Figure 2D). ATP effectively outcompeted RNA binding in a concentration-dependent manner, while ADP was found to be a poor competitor, indicating that the pocket can accommodate up to 3 phosphate groups and that NTPs may play a role in RNA ligand turnover. The arrangement of ADP and a 5′-monoPi RNA accessing the binding pocket from opposite sides was reminiscent of the inverted 5′ m^7^G cap structure of mRNA. Therefore, we investigated whether an m^7^G cap analogue could access hnRNPUL1’s substrate pocket. Unlabeled GTP effectively displaced the bound radioactive GTP, whereas the m^7^G cap analog did not, indicating that hnRNPUL1 is unlikely to accommodate a 5′ cap structure in its SPRY-dPNK pocket (Figure 2E, left). As a control, m^7^G efficiently displaced GTP from the cap-binding protein CBP20 in this type of assay (Figure 2E, right).

### Reactivation of the hnRNPUL1 dPNK domain

**Figure 3:**
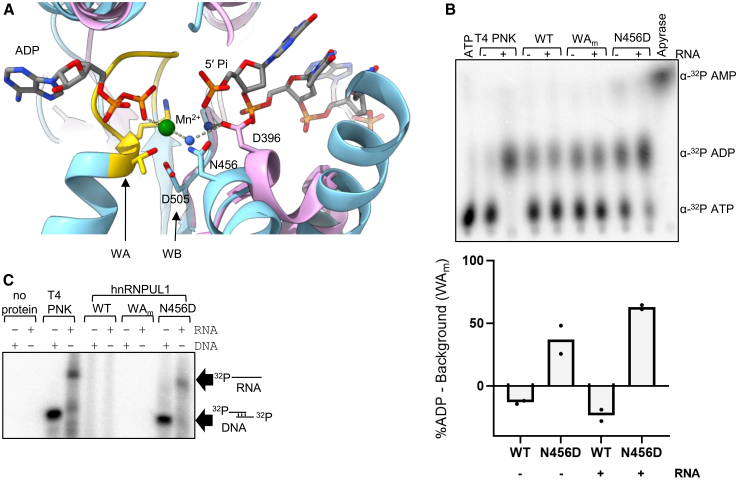
hnRNPUL1 is a dead polynucleotide kinase (A) Structural comparison between the hnRNPUL1 AlphaFold3 model (blue) and ligand-bound PNKP (violet, PDB: 3ZVN). Side chains of WA (gold) and WB motifs, as well as the catalytic Asp396 (PNKP) and Asn456 (hnRNPUL1) are shown in stick form. Dashed lines denote hydrogen bonds. (B) Thin-layer chromatography (TLC) assay measuring α-^32^P ADP released by mutants of full-length hnRNPUL1. T4 PNK is a positive control for ADP migration, while apyrase is a control for AMP migration. Inset: Quantification of the percentage of α-^32^P ADP generated above background (WA_m_ conditions) for two replicates, with the bar showing the mean value. (C) Kinase assay using full-length hnRNPUL1 variants, γ-^32^P ATP and RNA or dsDNA oligonucleotide substrates with free 5′ ends and 5′ overhangs. T4 PNK is used as positive control. See also Figure S3.

Despite the high structural homology between the dPNK domain of hnRNPUL1 and the equivalent domain of PNKP, we noted a key difference in the substrate-binding pockets of the two proteins. The catalytic D396 of PNKP is an asparagine at the structurally equivalent position in hnRNPUL1 (Figure 3A). Notably a D396N mutation in PNKP abolishes its polynucleotide kinase activity.^37^ Therefore, we speculated that reversing N456 to aspartate may restore the polynucleotide kinase activity to hnRNPUL1. An active polynucleotide kinase would be expected to hydrolyze ATP in the presence of a substrate nucleic acid. Remarkably, the N456D mutant displayed ATPase activity, which was considerably higher than the background activity seen with the WT and WA_m_ proteins (Figure 3B). While ATPase activity was observed for the N456D mutant, even in the absence of exogenous added RNA, it is possible that residual RNA co-purified with the proteins. The 5′-OH group provided by either co-purifying or exogenously added RNA may act as acceptor of the γ phosphates released by ATP hydrolysis. Strikingly, a kinase assay revealed that hnRNPUL1_N456D_ and T4 PNK were able to phosphorylate both RNA and dsDNA substrates with free 5′-OH ends (and overhangs), but hnRNPUL1 WT and WA_m_ lacked this ability (Figure 3C).

These results validate the structural model prediction and confirm that hnRNPUL1 is a dPNK in its natural form. Moreover, they demonstrate that hnRNPUL1 is among a few RNA-binding proteins that can specifically recognize the free 5′ end of an RNA, along with 5′–3′ exonucleases Xrn1, Xrn2, and DXO and polynucleotide kinases such as CLP1, Grc3, and NOL9.^38^^,^^39^^,^^40^^,^^41^^,^^42^ Despite the extensive sequence homology and predicted structural homology, hnRNPU could not be reactivated as a polynucleotide kinase by using a structurally equivalent N512D mutation (Figure S3). Moreover, hnRNPUL2, which naturally carries a glutamate at the position of N456 in hnRNPUL1, showed no polynucleotide kinase activity (Figure S3). Together, these results suggest that there are other differences, not readily detectable by the alignment of predicted structures of the dPNK domains of hnRNPU and hnRNPUL2, that prevent polynucleotide kinase activity.

### Mutations in the dPNK domain alter RNA-binding activity of full-length hnRNPUL1

**Figure 4:**
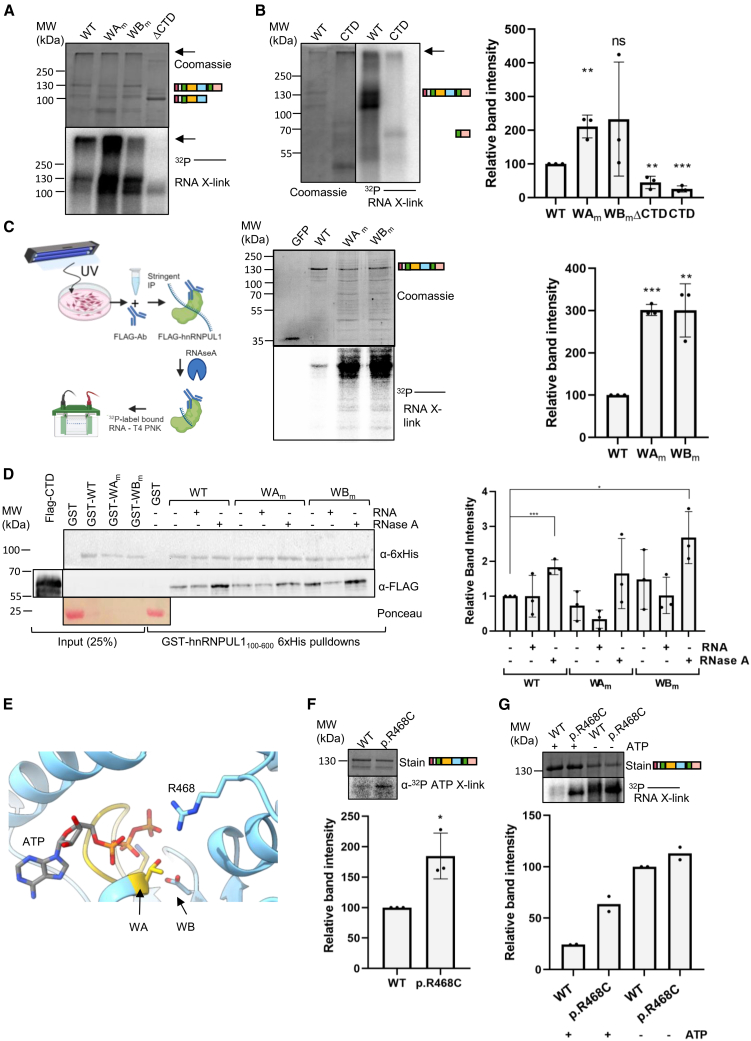
Nucleotide binding regulates interactions with RNAs (A and B) *In vitro* UV crosslinking of full-length hnRNPUL1 (WT, WA_m_, and WB_m_) and a ΔCTD truncation (A) or full-length hnRNPUL1 and the CTD (610–856) (B) to a 5′ ^32^P-labelled RNA oligonucleotide. Arrows point to high-molecular weight protein-RNA complexes trapped at the bottom of the wells. Inset: Quantification of band intensities relative to WT, with *p* values calculated using the unpaired *t* test (*n* = 3). (C) Left: Workflow for investigating *ex vivo* RNA binding of full-length hnRNPUL1 (WT, WA_m_, and WB_m_), with GFP used as negative control. HEK293T cells overexpressing N-terminal FLAG-tagged versions of those proteins were UV crosslinked, followed by stringent FLAG immunoprecipitation, limited RNase A digestion, and ^32^P end-labelling of the resulting RNA fragments on the beads and analysis by SDS-PAGE. Created in https://BioRender.com. Right: Results of *ex vivo* UV crosslinking of full-length hnRNPUL1 proteins to cellular RNA. Inset: Quantification of band intensities relative to WT, with *p* values calculated using the unpaired *t* test (*n* = 3). Data are represented as the mean ± SEM. (D) Pulldown of FLAG-tagged hnRNPUL1 CTD (610–856), using WT or mutant hnRNPUL1 SPRY-dPNK core (100–600). The bait protein carries an N-terminal GST tag and C-terminal 6xHis. Reactions were performed by incubation with RNase A (1.5 μM) or RNA oligonucleotide (70 nM) as indicated. Inset: Quantification of band intensities relative to untreated WT pulldown, with *p* value calculated using the unpaired *t* test (*n* = 3). Data are represented as the mean ± SEM. Statistical significance is shown by ns = *p* > 0.05, ∗ = *p* < 0.05, ∗∗ = *p* < 0.01, and ∗∗∗ = *p* < 0.001. (E) Detail of the hnRNPUL1 ligand pocket bound to ATP. Side chains of R468 and the WA (gold) and WB motifs are shown in stick form. (F) UV crosslinking of full-length hnRNPUL1 proteins to α-^32^P ATP. Inset: Quantification of mutant band intensity relative to WT, with *p* value calculated using the unpaired *t* test (*n* = 3). Data are represented as the mean ± SEM. (G) UV crosslinking of full-length hnRNPUL1 proteins to a 5′ ^32^P-labelled RNA oligonucleotide in the presence or absence of excess ATP. Inset: Quantification of band intensities relative to WT for two replicates, with the bar showing the mean value. See also Figure S4.

Next, we examined the RNA-binding effects of ligand pocket mutations on full-length hnRNPUL1 in the absence of NTPs. High-molecular weight protein-RNA complexes appeared after *in vitro* UV crosslinking (Figure 4A, arrows) and were absent from the ΔCTD condition, suggesting that the multiple disordered RNA-binding regions of the protein, especially the CTD (Figure S2), contribute to the formation of those large complexes. Consistent with this, the isolated CTD, which binds to RNAs, though less efficiently than the full-length protein, also had a tendency to form high-molecular weight complexes, which stuck in the well of the gel but were nevertheless capable of binding the RNA (Figure 4B). Deletion of the CTD dramatically reduced the RNA-binding activity (Figure 4A). This highlights the importance of RGG2 for the overall RNA-binding activity of the full-length protein. Strikingly, the full-length WA_m_ exhibited a substantial enhancement of RNA-binding activity compared with WT. This is in marked contrast to the RNA-binding activity with the isolated SPRY-dPNK domain (Figure 2B), where WA_m_ reduced the RNA-binding activity. The WB_m_ showed increased RNA-binding activity in all replicates, but the substantial variation between replicates led to the low statistical significance. Together, these data suggest that the dPNK domain may regulate the RNA-binding activity associated with the CTD.

We also investigated the RNA-binding activity of WT, WA_m_, and WB_m_ *ex vivo* by crosslinking-immunoprecipitation (CLIP), using FLAG-tagged versions, which allowed stringent immunoprecipitation of protein-RNA complexes, limited on-bead RNase A trimming and ^32^P end-labelling, and visualization by SDS-PAGE (Figure 4C, left). On the basis of eCLIP data from the ENCODE project,^24^ we expect hnRNPUL1 to crosslink with a broad range of RNAs in this assay including protein coding and long non-coding RNAs. In this assay, both WA_m_ and WB_m_ showed drastic enhancement of the RNA-binding activity compared with WT (Figure 4C, right). Both mutants also displayed additional protein bands, which persisted through high-salt (1 M NaCl) washes and produced a signal on the autoradiogram, which may correspond to proteolytic fragments of hnRNPUL1 or tightly interacting proteins. Overall, these results indicate that WA_m_ and WB_m_ enhance RNA interactions involving the CTD.

A number of proteins, such as the mRNA export factors Alyref^43^ and Nxf1,^44^ which have arginine-rich RNA-binding peptides within IDRs, form intramolecular interactions with their adjacent folded domains. In the case of Nxf1, the intramolecular interaction suppresses its RNA-binding activity. To investigate whether hnRNPUL1 CTD might form an intramolecular interaction, we assayed for an interaction between GST-hnRNPUL1 (aa 100–600), which includes regions of IDR1, along with the SPRY-dPNK domains, and the CTD (Figure 4D). We used this construct because we found that a more truncated SPRY-PNK domain produced in *E. coli* was poorly expressed with limited solubility. GST-hnRNPUL1 (aa 100–600) bound the CTD, indicating an intramolecular interaction. This interaction was not blocked by the addition of exogenous RNA. However, the CTD or the GST-hnRNPUL1 (aa 100–600) may have co-purified with host RNA. Therefore, we carried out the pulldown assay in the presence of RNase A and observed a robust increase in the amount of CTD pulled down with GST-hnRNPUL1 (aa 100–600).This was also the case with the GST fusion carrying WB_m_. This indicates that RNA binding and the intramolecular interaction are mutually exclusive. Thus, we considered that the WA_m_ and WB_m_ dPNK domains may release the CTD, leading to the increased RNA binding observed *in vivo* (Figure 4C). However, this was not readily detectable using a pulldown assay (Figure 4D), and, therefore, a more subtle conformational change may be responsible.

### Rare hnRNPUL1 coding variants in a cohort of ALS patients

The earlier identification of a rare coding variant of hnRNPUL1 in an ALS patient^18^ led us to screen cohorts of familial (*n* = 1,022, ALS variant server) and sporadic (*n* = 4,366)^45^ ALS patients for additional rare pathogenic coding variants within hnRNPUL1, which may contribute to the disease. We defined “rare” based on the minor allele frequency of <1% in population databases^46^ and “pathogenic” based on the combined annotation-dependent depletion score of >10.^47^ Twenty-one heterozygous variants identified using these criteria are shown (Figure S4A and Table S1). Notably, variant p.R541∗ involved a severe truncation, suggesting the loss of hnRNPUL1 function, whereas the effects of other variants were unclear. While some variants we identified in ALS patients have also been identified within the gnomAD^48^ database of human exome and genome sequencing data, their frequency in the general population is < 1 per 10,000 individuals. Despite this, the identification of hnRNPUL1 variants in ALS patients does not conclusively demonstrate their role in disease causation.

We investigated the effects of the p.R468C variant on ligand binding to the full-length protein. The variant amino acid, located near the ATP-binding pocket (Figure 4E), showed enhanced ATP binding compared with WT (Figure 4F). In the absence of ATP, RNA binding was found to be unaffected between full-length p.R468C and WT (Figure 4G, right 2 lanes), as seen with the isolated SPRY-dPNK domain (Figure 2B). However, in the presence of ATP, p.R468C bound RNA considerably better than the WT protein (Figure 4G, left 2 lanes) and so behaved in a similar manner to WB_m_. p.R468C lies within the flexible loop modeled to be involved in an ATP-induced conformational change (Figures 1G, S1C, and S1F). The AlphaFold3 model predicts that the mutation Arg→Cys would ablate the hydrogen-bonding network of the side chain (Figure S4B), which may perturb the motion of that region, altering the capacity of the protein to bind both ATP and RNA. Together, these data demonstrate that mutations and a variant that disrupt the hydrogen-bonding network within the dPNK domain (WA_m_, WB_m_, and p.R468C), potentially triggering conformational changes, all result in enhanced RNA binding, irrespective of whether the mutations/variant enhance or reduce NTP binding.

We assessed the potential structural impact of other hnRNPUL1 variants observed in ALS patients within the SPRY-dPNK core compared with the WT model (Figure S4). Of the 9 missense variants, only 3 were predicted to fully ablate hydrogen-bonding networks: p.N264S, p.S287A, and p.R468C. Variants p.S230T, p.S249N, and p.R357Q were predicted to result in one less hydrogen bond, while p.I580T was conversely predicted to introduce an additional side-chain to main-chain interaction with p.V530. The remaining two missense variants, p.P395S and p.D588Y, did not contribute to side-chain interactions in the missense or WT models, and their impact on hnRNPUL1 function remains to be determined.

### The dPNK domain governs hnRNPUL1 homotypic versus heterotypic interactions

**Figure 5:**
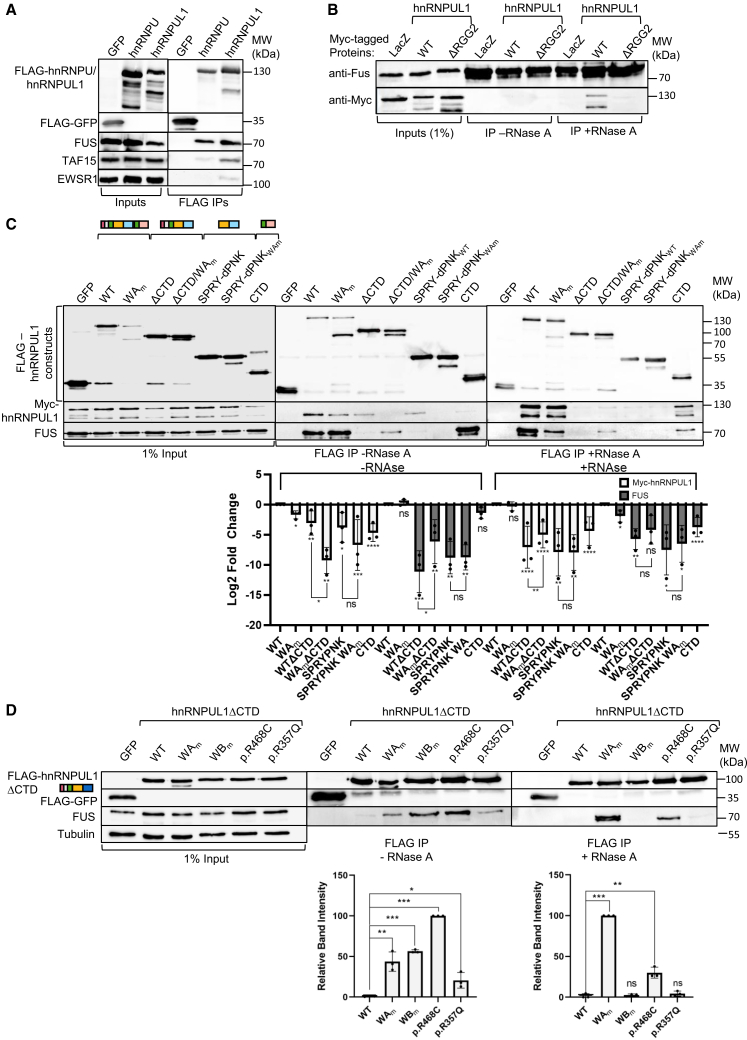
The dPNK domain governs homotypic vs. heterotypic interactions (A–D) Western blot analyses. (A) FLAG-hnRNPU/UL1 and FLAG-GFP immunoprecipitations from FLP-In cell lines with stable expression of the FLAG-tagged constructs probing for interactions with the FET protein family. (B) FUS immunoprecipitations from HEK293T cells expressing myc-tagged hnRNPUL1 mutants and lacZ. (C) FLAG immunoprecipitations from HEK293T cells co-transfected with Myc-hnRNPUL1 (full length) and FLAG-tagged GFP or hnRNPUL1 constructs as indicated. Inset: Quantification of log2 fold change of IP signal relative to full-length WT conditions, with *p* value calculated using the unpaired *t* test (*n* = 3). Data are represented as the mean ± SEM. (D) FLAG immunoprecipitations from HEK293T cells transfected with GFP or mutant FLAG-hnRNPUL1_ΔCTD_. RNase A was added to cell lysates prior to IPs as indicated. Inset: Quantification of IP signal relative to hnNRPUL1 ΔCTD conditions, with *p* value calculated using the unpaired *t* test (*n* = 3). Data are represented as the mean ± SEM. Statistical significance is shown as ns = *p* > 0.05, ∗ = *p* < 0.05, ∗∗ = *p* < 0.01, and ∗∗∗ = *p* < 0.001.

Several mass spectrometry studies have identified hnRNPUL1 as a binding partner for the FET (FUS, EWS, and TAF15) family of proteins,^15^^,^^49^^,^^50^^,^^51^ which are mutated in ALS patients.^52^ A co-immunoprecipitation experiment in the presence of RNase A confirmed a robust protein-protein interaction with FET proteins for both hnRNPUL1 and the related protein hnRNPU (Figure 5A). To explore the interaction between FUS and hnRNPUL1 in a reciprocal way, we immunoprecipitated FUS in the presence and absence of RNase A to identify protein-protein interactions (Figure 5B). In the absence of RNase A, an FUS immunoprecipitate did not exhibit readily detectable levels of hnRNPUL1; thus, in steady state, only a small proportion of FUS was associated with hnRNPUL1. However, in the presence of RNase A, hnRNPUL1 was readily detectable in an FUS immunoprecipitate, and this interaction was dependent on the presence of the RGG2 domain in hnRNPUL1. We infer from these results that the RGG2 domain of hnRNPUL1 forms mutually exclusive interactions with RNA and FUS. FET proteins are known to form biomolecular condensates *in vivo*, and hnRNPUL1 is also predicted to phase separate^14^. Therefore, it is possible that the observed co-immunoprecipitation of hnRNPUL1 with FET proteins is indirect by virtue of purification of a biomolecular condensate, rather than a direct protein-protein interaction between hnRNPUL1 and the FET proteins. Nevertheless, such interactions are not dependent on RNAs.

Many RNA-binding proteins including hnRNPU form both homotypic and heterotypic interactions.^53^^,^^54^ To examine the interplay between these interactions, we immunoprecipitated FLAG-hnRNPUL1 in cells expressing Myc-hnRNPUL1 while also screening for interactions with FUS (Figure 5C). In the absence of RNase, a hnRNPUL1 homotypic interaction was readily detectable with WT, WA_m_, and ΔCTD mutants, together with the isolated SPRY-dPNK domain. However, this homotypic interaction was disrupted when the WA_m_ mutation was combined with ΔCTD. The isolated CTD showed a very weak interaction with full length Myc-hnRNPUL1. On screening for heterotypic interactions with FUS, a robust interaction was detected with both WT and WA_m_ but neither with the ΔCTD mutant nor with the isolated SPRY-dPNK domain. Strikingly, the combination of the WA_m_ and ΔCTD mutations restored binding to FUS, albeit at significantly reduced levels compared with full-length WT and WA_m_. The switch from homotypic (ΔCTD) to the heterotypic (ΔCTD/WA_m_) FUS interaction driven by the WA_m_ mutation reveals that the SPRY-dPNK domain regulates homotypic versus heterotypic interactions. Because a key domain involved in FUS interactions is the hnRNPUL1 CTD (Figure 5C), these data are consistent with the idea that the dPNK domain regulates the activity of the CTD. However, given that the ΔCTD/WA_m_, but not the isolated SPRY-dPNK domain, could also bind FUS, it can be inferred that the N-terminal regions, including the SAP and IDR1, also contribute to FUS interactions and that these are also regulated by the SPRY-dPNK domain. We further investigated homotypic versus heterotypic interactions in the presence of RNase to discriminate between direct and RNA-bridged interactions. While the homotypic interactions with WT and WA_m_ mutants were preserved, interactions with other regions of hnRNPUL1 were significantly reduced, indicating that RNA stabilized these interactions (Figure 5C). The exception to this was the isolated CTD, which still formed a substantial homotypic interaction with full-length hnRNPUL1, consistent with the intramolecular interaction observed earlier between the CTD and aa 100–600 (IDR1-SPRY-dPNK) (Figure 4D).

During the course of these experiments, we noted that FLAG-hnRNPUL1 constructs carrying the WA_m_ mutation (Figure 5C, top) presented discrete truncations, which were absent from the equivalent WT constructs. For example, full-length WA_m_ has a prominent discrete C-terminal truncation, with a size very similar to the ΔCTD construct. These data, combined with the observation that WT hnRNPUL1 has no detectable ATPase or polynucleotide kinase activity, suggest that NTP binding serves to stabilize the SPRY-dPNK fold. The disruption of NTP binding by WA_m_ and the accompanying destabilization of the fold may trigger release of the flanking IDRs, making them more susceptible to proteolysis, leading to the discrete truncated proteins observed.

We further assessed the impact of SPRY-dPNK domain mutations and variants identified in ALS patients on interactions with FUS by using the ΔCTD construct, which is acutely sensitive to mutations in the SPRY-dPNK domain for such interactions (Figure 5D). In the absence of RNase, each mutant/variant showed an enhanced interaction with FUS compared with the WT ΔCTD protein, with WB_m_ and p.R468C showing the strongest interactions. Strikingly, in the presence of RNase, WA_m_ bound FUS better than the other mutants/variants. Notably, the two variants that more strongly bound ATP—WB_m_ and p.R468C—showed substantially reduced FUS interaction relative to WA_m_ in the presence of RNase, as did the p.R357Q variant lying within the SPRY domain, indicating that these interactions were at least partially bridged by RNA. In contrast, the WA_m_ interaction with FUS was enhanced following RNase treatment. Because the isolated SPRY-dPNK domain failed to bind FUS (Figure 5C), we conclude that the ability of the ΔCTD constructs to bind FUS in these assays is mediated by the SAP-IDR1 regions of hnRNPUL1. This activity is regulated by the SPRY-dPNK domain, and mutations/variants in this domain alter this regulatory activity.

## Discussion

**Figure 6:**
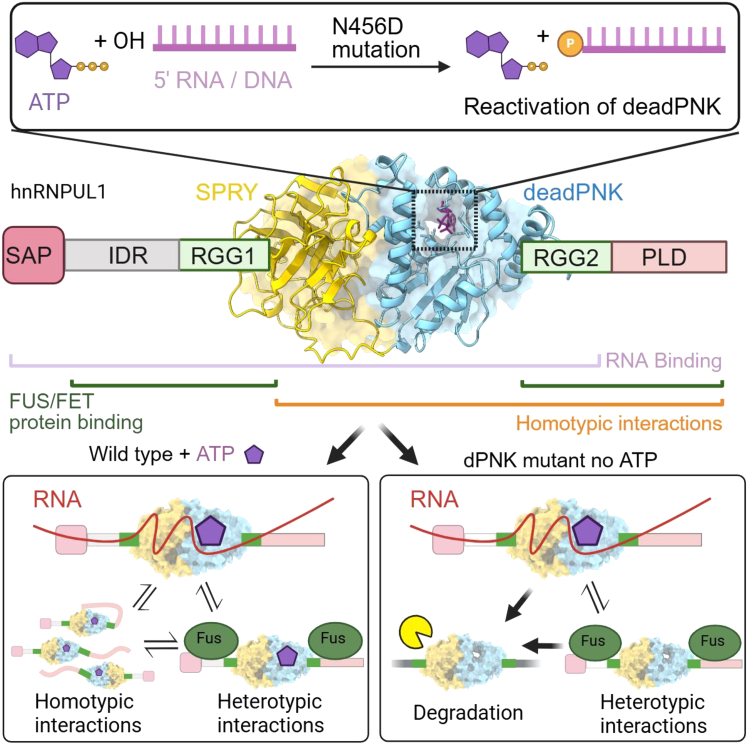
A model for hnRNPUL1 activities Schematic of the protein structure is shown, with the ligand-binding cleft highlighted, and the activity of the N456D mutant as a polynucleotide kinase shown above. Domains involved in protein and RNA interactions are highlighted below the domain schematic. Binding activities in the presence and absence of ATP or in a dPNK mutant unable to bind ATP are summarized in the two boxes below the domain schematic. Created in https://BioRender.com.

We have shown that hnRNPUL1 harbors a central tightly juxtaposed SPRY-dPNK domain fold, which can bind, but not hydrolyze, NTPs. However, ATP binding significantly altered the intrinsic tryptophan fluorescence, as previously observed for hnRNPU,^54^ suggesting a potential conformational change on nucleotide binding. Such a conformational change is observed in the structurally related kinase domain of mammalian PNKP (Figure S1F) on substrate binding. Mutations that disrupt NTP binding have a dramatic effect on the ability of hnRNPUL1 to bind itself, other proteins, and RNAs, indicating a central role for nucleotide binding in regulating its activity. The activities of hnRNPUL1 are summarized in a model (Figure 6). The ability to regulate activity through nucleotide binding, while not directly coupled to a nucleotide hydrolysis cycle, is not unique to hnRNPUL1. Yeast Clp1 binds but does not hydrolyze ATP,^55^ yet ATP binding regulates its interaction with its essential partner Pcf11 in 3′ end processing.^56^ As the intranuclear concentration of ATP is very high, it is not clear how nucleotide binding to hnRNPUL1 might be regulated *in vivo,* though it may be coupled with RNA interactions (Figure 2) or altered in response to energy starvation. Alternatively, ATP may always be bound and simply act as a co-factor to maintain the structure and stability of the dPNK domain and flanking disordered regions.

Strikingly, full-length hnRNPUL1 purified directly from human cells binds ATP with >200-fold higher affinity than that reported for hnRNPU.^53^ However, we noted that the earlier study utilized a fragment of hnRNPU lacking the tightly juxtaposed SPRY domain. Whether the truncation of hnRNPU led to partial disruption of the NTP-binding fold or whether hnRNPU genuinely binds ATP with such lower affinity is currently not clear. Comparison of the sequences and predicted structures (Figures 2 and S2) revealed substantial homology and no obvious reason for differences in the nucleotide-binding affinity based on their ligand-binding clefts.

While a mutation that disrupted nucleotide binding (WA_m_) led to enhanced interactions with proteins such as FUS and RNA via both IDR1 and the CTD, the WB_m_ and p.R468C mutant/variant, which bind ATP better than the WT protein, also showed dramatically enhanced RNA binding and IDR1-mediated interactions with FUS. A common feature of all these mutants is disruption of the hydrogen-bonding network within the dPNK fold. The predicted conformational changes within the dPNK domain associated with this disruption led to rampant protein or RNA ligand binding via the flanking IDR1 and CTD. Therefore, a major function of the dPNK domain may simply be to sequester the flanking IDR1 and CTD regions and prevent excessive association with either RNA or other proteins, with ATP stabilizing the dPNK fold to maintain this activity. The use of a central folded domain to sequester and regulate the activity of flanking RGG boxes containing IDRs is not unique to hnRNPUL1. Two mRNA export factors, Alyref^43^ and Nxf1,^44^ also exhibit this activity. Moreover, FUS forms head-to-tail interactions driven by cation-π interactions, which, in turn, is driven by a C-terminal IDR containing RGG peptides and an aromatic rich N-terminal domain.^57^ Thus, self-regulation of RGG domains within IDRs may be more widespread, maintaining the correct balance of ligand interactions as well as stabilizing the IDRs, protecting them from proteolytic attack in their ligand-free state. Consistent with this latter idea, we observed significant degradation on hnRNPUL1 in a variety of truncations carrying the WA_m_ mutation (Figure 5C).

The dPNK domain of hnRNPUL1 has the ability to bind the 5′ end of uncapped RNAs. Such RNAs are produced during 3′ cleavage of pre-mRNAs, and the downstream RNA is subsequently degraded by Xrn2 to drive RNA Pol II transcription termination.^58^ Furthermore, hnRNPUL1 was previously identified in a purified 3′ processing complex.^59^ Therefore, it is conceivable that hnRNPUL1 may antagonize the activity of Xrn2 *in vivo,* though this remains to be confirmed. Alternatively, rather than docking an RNA 5′ end, the dPNK domain, together with the associated SPRY domain, may act as a more general RNA-binding domain. In support of this, mass spectrometry approaches have identified peptides within the SPRY-dPNK region that bind RNA (Figure S2), and a recent unbiased mass spectrometry study looking for proteins binding RNA G-quadruplexes identified the dPNK domain of hnRNPUL1.^60^ Within FUS, sequence-independent unstructured RNA-binding RGG boxes lie adjacent to two folded domains, which bind RNA selectively—the RNA recognition and zinc finger motifs. The RGG boxes play a role in destabilizing RNA structures and dramatically increase the overall FUS RNA-binding affinity.^61^ By analogy, it is conceivable that hnRNPUL1 uses its dPNK domain to bind structured RNAs such as RNA G-quadruplexes, with the adjacent RGG boxes enhancing the RNA-binding affinity. This may account for why hnRNPUL1 binds ALS-associated C9orf72 repeat expansion RNA,^17^ which is known to form RNA G quadruplexes.^62^

A significant number of the hnRNPUL1 variants we observed were within the two IDRs, IDR1 and the CTD, and this is observed in ALS genes such as *FUS* and *TDP43*.^63^ The formation of biomolecular condensates through intrinsically disordered domains is a common feature of RNA-binding proteins, and this property influences their activity in splicing regulation through modulation of both protein and RNA interactions.^64^ Therefore, the multiple IDR1 and CTD variants in hnRNPUL1 may alter its activity in alternative splicing regulation.^7^^,^^8^ Similarly, we have shown that mutations/variants within the folded SPRY-dPNK region alter RNA and protein binding for hnRNPUL1, which again are likely to impact its activity in RNA splicing, DNA damage repair, and, ultimately, cellular fitness.

### Limitations of the study

As we do not have access to individual-level genotyping from sufficient numbers of non-ALS individuals, we were unable to perform a statistically significant gene burden test to establish that the hnRNPUL1 variants we have observed are ALS specific. However, we noted that they occur in <1 per 10,000 individuals with the gnomAD database but at >10-fold higher frequency in the ALS cohorts we examined. Furthermore, we have not established the RNA-binding specificity of the individual RNA-binding domains of hnRNPUL1. For example, it is not clear whether the dPNK domain binds to the 5′ end of an RNA in the manner that PNKP would *in vivo*, or whether, on losing polynucleotide kinase activity, the dPNK domain, along with the adjacent SPRY domain, forms a more general RNA-binding domain*.* Further *in vivo* studies using isolated domains and measuring RNA-binding activity using CLIP approaches may resolve these questions.

## Resource availability

### Lead contact

Further information and requests for reagents may be directed to and will be fulfilled by the lead contact, Stuart A Wilson (stuart.wilson@sheffield.ac.uk).

### Materials availability

Plasmids and other reagents are available from the lead contact.

### Data and code availability

• Original uncropped phosphor images and western blot images have been deposited at Mendeley (https://doi.org/10.17632/rh3prrcskn.1) and are publicly available as of the date of publication.
• This paper does not report original code.
• Any additional information required to reanalyze the data reported in this paper is available from the lead contact upon request.

## Acknowledgments

We thank Dr. Phil Mitchell for insightful conversations and experimental help with the TLC assay in Figure 3B. We thank Dr. Pat Baker for helping in generating the closed and open hnRNPUL1 structures in Figure S1F. S.A.W. acknowledges support from the 10.13039/501100000268Biotechnology and Biological Sciences Research Council UK (10.13039/501100000268BBSRC) grants BB/W000172/1, BB/N005430/1, and BB/N014839/1. C.V.A. and I.D.Y. acknowledge support from the 10.13039/501100000268BBSRC White Rose Doctoral training program grant BB/M011151/1. E.A.-M. acknowledges support from 10.13039/501100003141CONACYT-Mexico.

## Author contributions

C.V.A., A.L., P.D., L.G., E.A.-M., A.G.R.W, and I.D.Y. carried out the experiments and analyzed data; J.C.-K. identified the ALS patient mutations in hnRNPUL1; P.J.S., I.M.S., and S.A.W. supervised the work; C.V.A. and S.A.W. wrote the manuscript with contributions from all authors.

## Declaration of interests

The authors declare no competing interests.

## STAR★Methods

### Key resources table

**Table:** 

REAGENT or RESOURCE	SOURCE	IDENTIFIER
**Antibodies**		

Mouse monoclonal anti-FLAG tag (clone M2)	Millipore, Sigma	Cat# F3165; RRID:AB_259529
Mouse monoclonal anti-6xHis tag HRP-conjugate (clone GT359)	Abcam	Cat# ab184607; RRID:AB_2868537
Mouse monoclonal anti-Tubulin (clone B-5-1-2)	Millipore, Sigma	Cat# T5168; RRID:AB_477579
Rabbit polyclonal anti-hnRNPUL1	This paper	N/A
Rabbit polyclonal anti-FUS	Novus Biologicals	Cat# NB100-565; RRID:AB_523761
Rabbit monoclonal anti-TAF15 [clone EPR9197(B)]	Abcam	Cat# ab134916; RRID:AB_2614922
Rabbit polyclonal anti-EWSR1	Bethyl Laboratories	Cat# A300-417A; RRID: AB_420957
Mouse monoclonal anti-hnRNPU (clone 3G6)	Abcam	Cat# ab10297; RRID:AB_297037
Mouse monoclonal anti-Myc tag antibody (clone 9E10)	Abcam	Cat# ab32; RRID:AB_303599
Goat polyclonal anti-mouse IgG (H + L) HRP-conjugate	Promega	Cat# W4021; RRID: AB_430834
Goat polyclonal anti-rat IgG (H + L) HRP-conjugate	ThermoFisher	Cat# 62–9520; RRID: AB_2533965
Goat polyclonal anti-rabbit IgG (H + L) HRP-conjugate	Promega	Cat# W4011; RRID:AB_430833

**Bacterial and virus strains**		

*Escherichia coli* DH5α	Fisher Scientific	Cat# 18-265-017
*Escherichia coli* BL21 DE3	Fisher Scientific	Cat# 10749734

**Chemicals, peptides, and recombinant proteins**		

Apyrase	Millipore, Sigma	Cat# A6132; CAS: 9000-95-7
Blasticidin	Melford	Cat #B12150–0.1; CAS: 3513-03-9
Hygromycin B	Invitrogen	Cat # 10687010; CAS: 31282-04-9
Penicillin/Streptomycin	Gibco	Cat # 15140122
Tetracycline	Sigma	Cat # 87128-25G; CAS: 60-54-8
Zeocin	ThermoFisher	Cat #R25005
Actinomycin D	Millipore Sigma	Cat# A1410; CAS: 50-76-0
Fetal bovine serum (FBS), Tetracycline-free	Biosera	Cat# FB-1001T/500
Trypsin EDTA	Biosera	Cat# LM-T1705/500
TurboFect Transfection Reagent	ThermoFisher	Cat# R0533
Dulbecco’s Modified Eagle Medium (DMEM)	VWR	Cat# L0101-500
3xFLAG peptide	Millipore, Sigma	Cat# F4799
Lipofectamine 2000	Invitrogen	Cat# 11668027
TLC PEI cellulose sheets	Macherey-Nagel	Cat# 801063
cOmplete EDTA-free Protease Inhibitor Cocktail	Roche	Cat# 11873580001
Proteinase K	Millipore, Sigma	Cat# 3115879001
RNase A	Millipore, Sigma	Cat# 10109169001
GlycoBlue™ Coprecipitant	ThermoFisher	Cat# AM9515
RiboSafe RNase Inhibitor	Meridian Bioscience	Cat# BIO65028
TURBO DNase	Invitrogen	Cat# AM2239
α-32P ATP	PerkinElmer/Revvity	Cat# BLU503H250UC; CAS: 987-65-5
ɣ-32P ATP	PerkinElmer/Revvity	Cat# BLU502Z250UC; CAS: 987-65-5
α-32P GTP	PerkinElmer/Revvity	Cat # BLU506H250UC; CAS: 36051-31-7
m7GP3G (monomethyl cap analog)	Jena Bioscience	Cat# NU-852S: CAS: 62828-64-2
AMP-PNP	Millipore, Sigma	Cat# NC1366768; CAS: 25612-73-1
GMP-PNP	Millipore, Sigma	Cat# G0635; CAS: 148892-91-5
ADP	Millipore, Sigma	Cat# A-5285; CAS: 72696-48-1
GTP	Millipore, Sigma	Cat# G8877; CAS: 36051-31-7
T4 PNK (3′ phosphatase minus)	New England Biolabs	Cat# M0236S
Glutathione Sepharose 4B	GE Healthcare	Cat# 17-0756-01
Costar Spin-X Centrifuge Tube Filters	Corning	Cat# 8161

**Critical commercial assays**		

Gibson assembly cloning kit	New England Biolabs	Cat# E5510S
Q5 Site-Directed Mutagenesis Kit	New England Biolabs	Cat# E0554S

**Deposited data**		

Uncropped gel images	This paper	https://doi.org/10.17632/rh3prrcskn.1

**Experimental models: Cell lines**		

HEK293T	ECACC	Cat# 12022001
Flp-In T-Rex 293	Invitrogen	Cat# R78007
Tetracycline-inducible FLAG-hnRNPU Flp-In 293	This paper	N/A
Tetracycline-inducible FLAG-hnRNPUL1 Flp-In 293	This paper	N/A

**Oligonucleotides**		

RNA sequence: mono-Pi oligo: GCUUGCAUGCCUGCAGGCC AGCCUCAAUCACAUC	This paper	N/A
DNA oligo sequence: kinase substrate fw: CTTCAGGATGGAAG	This paper	N/A
DNA oligo sequence: kinase substrate rv: GTGACCATCTTCCA	This paper	N/A

**Recombinant DNA**		

pPGKFLPobpA Flp recombinase	Addgene	Cat# 13793
pcDNA™5/FRT Mammalian Expression Vector	Invitrogen	Cat# V601020
pcDNA5-FRT 3xFLAG hnRNPU	This paper	N/A
pcDNA5-FRT 3xFLAG hnRNPUL1	This paper	N/A
pcDNA5-FRT 3xFLAG hnRNPUL1 WA_m_	This paper	N/A
pcDNA5-FRT 3xFLAG hnRNPUL1 WB_m_	This paper	N/A
pcDNA5-FRT 3xFLAG hnRNPUL1 N456D	This paper	N/A
pcDNA5-FRT 3xFLAG hnRNPUL1 R468C	This paper	N/A
pcDNA5-FRT 3xFLAG hnRNPUL1 ΔCTD	This paper	N/A
pcDNA5-FRT 3xFLAG hnRNPUL1 ΔCTD WA_m_	This paper	N/A
pcDNA5-FRT 3xFLAG hnRNPUL1 ΔCTD WB_m_	This paper	N/A
pcDNA5-FRT 3xFLAG hnRNPUL1 ΔCTD R468C	This paper	N/A
pcDNA5-FRT 3xFLAG hnRNPUL1 ΔCTD R357Q	This paper	N/A
pcDNA5-FRT 3xFLAG SPRY-dPNK	This paper	N/A
pcDNA5-FRT 3xFLAG SPRY-dPNK WA_m_	This paper	N/A
pcDNA5-FRT 3xFLAG SPRY-dPNK WB_m_	This paper	N/A
pcDNA5-FRT 3xFLAG SPRY-dPNK T507A	This paper	N/A
pcDNA5-FRT 3xFLAG SPRY-dPNK R516A	This paper	N/A
pcDNA5-FRT 3xFLAG SPRY-dPNK R468C	This paper	N/A
pcDNA5-FRT 3xFLAG RGG-PLD	This paper	N/A
pcDNA5-FRT 3xFLAG GFP	This paper	N/A
pcDNA3.1/myc-HisA	ThermoFisher	Cat# V80020
pcDNA3.1/myc-HisA- hnRNPUL1	This paper	N/A
pcDNA3.1/myc-HisA- hnRNPUL1 ΔRGG2	This paper	N/A
pcDNA3.1/myc-HisA- LacZ	This paper	N/A
pGEX-6P-1	Amersham	Cat# 27-4597-01
pGEX-6P-1 hnRNPUL1_100-600_ 6xHis	This paper	N/A
pGEX-6P-1 hnRNPUL1_100-600_ WA_m_ 6xHis	This paper	N/A
pGEX-6P-1 hnRNPUL1_100-600_ WB_m_ 6xHis	This paper	N/A

**Software and algorithms**		

ChimeraX 1.9	Meng et al.^65^	https://www.rbvi.ucsf.edu/chimerax/RRID:SCR_015872
GraphPad Prism 10.5.0	NA	https://www.graphpad.com/RRID:SCR_002798
AlphaFold v3.0	Abramson et al.^28^	https://alphafoldserver.com/about RRID:SCR_025885
BioRender	NA	https://BioRender.com RRID:SCR_018361
ImageJ	Schneider et al.^66^	https://imagej.net/ij/RRID:SCR_003070
Image Lab	NA	http://www.bio-rad.com/en-us/sku/1709690-image-lab-software RRID:SCR_014210

### Experimental model and study participant details

#### Cell lines

The HEK293T cell line was purchased from the European Collection of Authenticated Cell Cultures (ECACC) and the Flp-In-T-Rex 293 cell line was purchased from Invitrogen. Both cell lines were routinely tested for Mycoplasma contamination but were not further authenticated following purchase. Cell lines were grown at 37 °C with 5% CO_2_ in Dulbecco’s modified Eagle medium (DMEM) with 10% fetal bovine serum (FBS).

### Method details

#### Cloning and mutagenesis

hnRNPUL1 full length and truncation constructs, as well as GFP, were cloned into the pcDNA 5/FRT plasmid with an N-terminal 3xFLAG tag sequence using the Gibson assembly method according to the manufacturer’s instructions. Separately, the hnRNPUL1 region 100–600 was cloned by Gibson assembly into the pGEX-6P-1 vector with an N-terminal GST tag and a C-terminal hexahistidine tag. Point mutations were introduced using the Q5 Site-Directed Mutagenesis kit following the manufacturer’s instructions. All plasmids generated in this study were validated by Sanger sequencing.

#### Generation of FlpIN FLAG-hnRNPUL1 stable Cell lines

FlpIn-293 (10,000 cells) were seeded in a 6-cm dish and cultured in Tet-free FBS DMEM for 24 h at 37 °C before transfection with 2.4 μg FRT vector and 3.6 μg FlpIn recombinase (pPGKFLPobpA) using Turbofect transfection reagent. Cells were split into two 10-cm dishes 48 h later. Selection medium (DMEM containing 15 μg/mL Blasticidin and 0.1 mg/mL Hygromycin) was supplied until individual colonies grew, which were transferred to wells in 24-well plates and expanded to allow screening via Western blotting. FLAG-tagged protein expression was induced for 48 h using tetracycline (1 μg/mL).

#### Transfection

Cells were seeded 24 h before transfection to achieve ∼70% confluency at the time of transfection. Lipofectamine 2000 was used to transfect cells at 5 μL/2.5 μg of DNA in a mixture of serum-free DMEM for a final volume of 1:10 to the dish volume. Cells were harvested 48 h later. For protein overexpression in mammalian cells, HEK293T were seeded in 10 cm dishes, similarly. Cells were transfected with a mixture of PEI:DNA of 3.5:1 (w/w) in serum-free DMEM for a final volume of 1:6 to the dish volume and harvested 48 h later.

#### Bacterial protein expression

*E.coli* BL21 cells were transformed with pGEX6P1 vectors harboring hnRNPUL1_100-600_ truncations were used to inoculate Terrific Broth at 37 °C at a 1:100 dilution from an overnight starter culture. At OD600 = 0.6, protein expression was induced with 1 mM IPTG and allowed to progress overnight at 18 °C.

#### Immunoblotting

Western blotting was performed on Amersham Protran nitrocellulose membrane with a Trans-Blot Turbo Transfer System. Proteins were transferred at 25 V, 1.3 mA for 22 min. Blots were incubated in TBST +5% milk for 1 h, then with primary antibody for 1 h, washed in 1x TBST 3 × 5 s and 3 × 5 min. Incubation (1 h) with secondary antibody (at 1:10,000) coupled to horseradish peroxidase (HRP) followed. Membranes were washed as before, placed in ECL detection reagent for 1 min and exposed in a Bio-Rad Chemidoc System.

#### Purification of FLAG-tagged proteins

HEK293T cells were lysed in 5 pellet volumes IP Lysis buffer (IPLB) (50 mM HEPES-NaOH pH 7.5, 100 mM NaCl, 0.5% Triton X-100, 1 mM EDTA pH 8.0, 10% Glycerol, 1 mM DTT, EDTA-free Protease Inhibitors) and nuclei were sheared by aspiration with a 26-gauge needle. hnRNPUL1 WA_m_ and WB_m_ lysates were digested with 500 U benzonase at 37 °C for 30 min. Lysates were centrifuged at 16,200x *g* for 5 min and supernatants transferred onto 100 μL FLAG agarose beads pre-washed with IPLB. Beads were incubated at 4 °C for 2 h, washed with 2 × 1 mL IPLB and treated with 4 μg RNase A at 37 °C for 30 min. Beads were washed with 3 × 1 mL IP wash buffer (IPLB +1 M NaCl) and once with 1 mL IPLB. Proteins were eluted in 300 μL IPLB +0.25 μg/μL 3xFLAG peptide at 4 °C. The *in vitro* experiments presented in Figures 1, 2, and 3, S3, 4A, 4B, 4E, and 4F utilised the FLAG-tagged proteins purified as described here.

#### RNA oligonucleotide labeling

An RNA oligonucleotide with 5′ hydroxyl was end-labelled with T4 PNK. The reaction contained 0.5 μM RNA, 16.7 μM ^32^P γ-ATP, 10 U T4 PNK in commercial T4 PNK buffer (NEB) and was incubated at 37 °C for 1 h, then RNA was separated on a 20% polyacrylamide +6 M urea gel in 0.5x TBE buffer (0.44 M Tris, 0.44 M Boric Acid, 1 mM EDTA, pH 8.0). The RNA was cut from the gel and crushed, then extracted by overnight soaking in 400 μL RNA gel extraction buffer (1 M NaCH_3_COO^−^, 1 mM EDTA). The supernatant was filtered through a Spin-X centrifuge tube and precipitated with 1 mL 100% ethanol and 5 μg glycogen. After 1 h at −20 °C, the RNA was centrifuged at 16,200x *g* for 30 min. The pellet was washed with 1 mL 75% ethanol, centrifuged at 16,200x *g* for 7 min, air dried and resuspended in 100 μL nuclease-free water.

#### NTP crosslinking and competition assays

##### ATP UV-crosslink

Proteins (0.3 μM) were mixed in NTP reaction buffer (NTP RB: 50 mM Tris-HCl pH 8.0, 100 mM NaCl, 10 mM MgCl_2_, 1 mM DTT) and 55 nM ^32^P γ-ATP. Reactions were incubated on ice for 15 min and UV-crosslinked on ice for 30 min at 254 nm. Crosslinked proteins were separated on an 8% gel by SDS-PAGE, stained and dried at 80 °C for 30 min. Dried gels were exposed on a phosphorimager screen, which was developed on a TyphoonFLA 7000 laser scanner.

##### NTP competition

Full-length hnRNPUL1 (1.6 μM) was mixed in NTP RB with 55 nM ^32^P γ-ATP and AMP-PNP or GMP-PNP non-hydrolysable competitors (final concentrations: 0.55, 5.5 and 27.7 μM). Reactions were mixed for 15 min on ice and UV-crosslinked for 30 min. Complexes were separated on a 10% gel by SDS-PAGE and processed as before.

##### GTP/m^7^G cap analog competition

hnRNPUL1 ΔCTD (1 μM) was mixed in NTP RB with 10 nM α-^32^P GTP and GTP or m^7^G cap analog competitors at 0.1, 1 and 10 μM. Samples were incubated at 37 °C for 15 min and UV-crosslinked on ice for 30 min. Complexes were separated on a 10% gel by SDS-PAGE and processed as before.

#### RNA UV-crosslinking and competition

##### RNA UV-crosslink

SPRY-dPNK or full-length proteins (0.5 μM) were mixed in RNA binding buffer (15 mM HEPES pH 7.9, 100 mM NaCl, 5 mM MgCl_2_, 0.2 mM EDTA, 0.05% Tween 20, 10% glycerol) with ∼25 nM 5′-labelled RNA oligonucleotide and 8 U RNAse inhibitor. Reactions incubated on ice for 10 min, then UV-crosslinked on ice for 15 min. Subsequently, proteins were separated on a 10% gel by SDS-PAGE and processed as before.

##### ATP/ADP competition

hnRNPUL1 ΔCTD (0.5 μM) was incubated in NTP RB with ∼25 nM 5′-labelled RNA oligonucleotide, 8 U RNase inhibitor and ATP or ADP competitors at 10, 100 and 1,000 μM. Reactions were incubated at 37 °C for 10 min and UV-crosslinked on ice for 15 min. Complexes were separated on a 10% gel by SDS-PAGE and processed as before.

##### *Ex vivo* RNA crosslinking

One 10-cm dish of HEK293T cells was transfected with each protein. Before harvesting, the cells were washed with PBS and UV-crosslinked on ice for 15 min. Purification of FLAG-tagged proteins proceeded as before, but without enzymatic treatments. On-bead RNase A digestion (8 U/condition) was performed at 37 °C for 10 min, followed by 3 washes in IPLB +1 M NaCl and RNA end-labelling with 3.3 nmol ^32^P γ-ATP and 1 U T4 PNK in T4 PNK RB for 10 min. Residual ATP and T4 PNK were removed with 3 washes in IPLB +1 M NaCl, followed by elution with 0.25 μg/μL 3xFLAG peptide, separation by SDS-PAGE and phosphorimaging.

#### Kinase assays and thin-layer chromatography

##### Kinase assays

Proteins (0.5 μM) were mixed in NTP RB with 0.5 μM dsDNA or an RNA oligonucleotide, carrying 5′ hydroxyl groups and 15 nM ^32^P γ-ATP. The T4 PNK control contained 10 U enzyme in T4 PNK RB instead. Reactions were incubated at 37 °C for 30 min and separated on a 12% 6 M urea polyacrylamide gel in 0.5X TBE. The gel was exposed on a phosphorimager screen for 1 h and developed as before.

##### TLC

ATP hydrolysis reactions were performed in NTP RB with 0.5 μM protein, 0.8 nM ^32^P γ-ATP and 0.5 μM RNA oligonucleotide (as specified). Reactions were incubated at 37 °C for 30 min and quenched with 0.25 M EDTA and 10 μL xylene cyanol. Proteinase K (20 μg) and 100 mM Tris pH 7.5 were added and proteins were digested for 30 min at 37 °C. T4 PNK and apyrase positive controls were performed differently. T4 PNK reactions were carried out in T4 PNK RB, ±0.5 μM RNA oligonucleotide, 0.8 nM ^32^P γ-ATP and 10 U T4 PNK. The apyrase control reaction contained 5 μg apyrase and 0.8 nM ^32^P γ-ATP in NTP RB. Reactions were incubated at 37 °C for 30 min, then quenched with 0.25 M EDTA and 20 μL xylene cyanol. PEI-cellulose plates were pre-run in 0.4 M phosphate buffer pH 5.5 and dried. Equal amounts of radioactivity were spotted on PEI plates and run in the same buffer, dried and exposed on a phosphorimager screen for 1 h and developed as before.

#### Fluorescence spectroscopy

SPRY-dPNK proteins were buffer-exchanged into 50 mM Tris pH 8.0, 100 mM NaCl. Measurements were carried out with 0.5 μM proteins in 50 mM Tris pH 8.0, 100 mM NaCl, 10 mM MgCl_2_, 5 mM DTT. Tryptophan fluorescence was measured with a Cary Eclipse fluorometer (Agilent), excitation wavelength = 280 nm and emission spectra = 300–400 nm with spectral resolution of 5 nm and photomultiplier set to high. Background fluorescence was measured from the reaction buffer and subtracted from the final results. Each protein was measured in apo form, then ATP was titrated into the solution. Final ATP concentrations tested were: 10, 25, 50, 74, 99, 246, 491, 735 and 978 nM. After each titration, proteins were incubated for 1 min at 37 °C and emission spectra recorded in triplicate. Five values around the emission peak (347–352 nm) were averaged for each spectrum and fluorescence quenching (ΔFluorescence) was calculated for each set of triplicate measurements. Curves were fitted in Graphpad Prism 8.3.0 corresponding to a One site– Specific binding equation.

#### GST-hnRNPUL1 pulldown

Bacterial cells were resuspended in 1 mL GST lysis buffer (1X PBS, 0.1% Tween 20) and sonicated (5 x [3s-ON/3s-OFF]) at 25% amplitude with a Fisherbrand Model 120 Sonic Dismembrator. Lysates were centrifuged at 16,200x *g* for 5 min and incubated with 120 μL GSH beads for 30 min at 4 °C. Beads were washed 3x with 1 mL GST lysis buffer, then split into 10 μL aliquots and mixed with purified FLAG-CTD in RB100 buffer (25 mM HEPES pH 7.5, 50 mM NaCl, 10 mM MgCl_2_, 1 mM DTT, 0.05% Triton X-100, 10% Glycerol) supplemented with RNase A (1.5 μM) or RNA oligonucleotide (70 nM) as indicated. Reactions were incubated at 37 °C for 30 min, supernatants washed with 2x 200 μL PBS and eluted with 2 bead volumes of 50 mM Tris pH 8.0, 40 mM GSH, 200 mM NaCl, 10% glycerol. Eluates were analyzed by SDS-PAGE and Western blotting.

#### FLAG-tagged Protein co-IP

FLAG-agarose (50 μL) was blocked by rotating overnight at 24 rpm and 4 °C in IPLB +1% BSA. Cells were lysed and supernatant loaded onto beads and incubated at 4 °C for 2 h. For RNase A-treated samples, 0.25 mg/mL RNase A was supplemented during the co-immunoprecipitation and washes before elution. Bound complexes were eluted in 60 μL IPLB +100 μg/mL 3xFLAG peptide by rotation at 4 °C for 1 h. Eluates were analyzed by SDS-PAGE and Western blotting, with input samples representing 0.1–0.5% of protein concentration loaded onto the beads. For FUS immunoprecipitations, 4 μg of FUS antibody was bound to 40 μL of Protein G Dynabeads and used similarly to FLAG-agarose in co-immunoprecipitation experiments but eluted using 3 M Arg.HCl pH 3.5 and neutralised with 1 M Tris-HCl pH 8.0.

#### Graphics generation

Structural models were analyzed and molecular models rendered using UCSF ChimeraX v1.9. Diagrams were drawn using BioRender.com.

### Quantification and statistical analysis

The construction of all graphs used Graphpad Prism v10.5.0. Error bars are SEM and individual data points are plotted. An unpaired *t* test was used to determine statistical significance between groups where *p* values are represented as follows: ns = *p* > 0.05, ∗ = *p* < 0.05, ∗∗ = *p* < 0.01 and ∗∗∗ = *p* < 0.001. *n* = the number of biological replicates. The specific statistics and details can be found in each figure legend.

## References

[1] Dreyfuss G., Matunis M.J., Piñol-Roma S., Burd C.G. (1993). hnRNP PROTEINS AND THE BIOGENESIS OF mRNA. Annu. Rev. Biochem..

[2] Dreyfuss G., Kim V.N., Kataoka N. (2002). Messenger-RNA-binding proteins and the messages they carry. Nat. Rev. Mol. Cell Biol..

[3] Gabler S., Schütt H., Groitl P., Wolf H., Shenk T., Dobner T. (1998). E1B 55-kilodalton-associated protein: a cellular protein with RNA-binding activity implicated in nucleocytoplasmic transport of adenovirus and cellular mRNAs. J. Virol..

[4] Bachi A., Braun I.C., Rodrigues J.P., Panté N., Ribbeck K., von Kobbe C., Kutay U., Wilm M., Görlich D., Carmo-Fonseca M., Izaurralde E. (2000). The C-terminal domain of TAP interacts with the nuclear pore complex and promotes export of specific CTE-bearing RNA substrates. RNA.

[5] Sharma S., Anand R., Zhang X., Francia S., Michelini F., Galbiati A., Williams H., Ronato D.A., Masson J.-Y., Rothenberg E. (2021). MRE11-RAD50-NBS1 Complex Is Sufficient to Promote Transcription by RNA Polymerase II at Double-Strand Breaks by Melting DNA Ends. Cell Rep..

[6] Polo S.E., Blackford A.N., Chapman J.R., Baskcomb L., Gravel S., Rusch A., Thomas A., Blundred R., Smith P., Kzhyshkowska J. (2012). Regulation of DNA-end resection by hnRNPU-like proteins promotes DNA double-strand break signaling and repair. Mol. Cell.

[7] Blackwell D.L., Fraser S.D., Caluseriu O., Vivori C., Tyndall A.V., Lamont R.E., Parboosingh J.S., Innes A.M., Bernier F.P., Childs S.J. (2022). Hnrnpul1 controls transcription, splicing, and modulates skeletal and limb development in vivo. G3 (Bethesda)..

[8] Vivori C., Papasaikas P., Stadhouders R., Di Stefano B., Rubio A.R., Balaguer C.B., Generoso S., Mallol A., Sardina J.L., Payer B. (2021). Dynamics of alternative splicing during somatic cell reprogramming reveals functions for RNA-binding proteins CPSF3, hnRNP UL1, and TIA1. Genome Biol..

[9] Ideue T., Adachi S., Naganuma T., Tanigawa A., Natsume T., Hirose T. (2012). U7 small nuclear ribonucleoprotein represses histone gene transcription in cell cycle-arrested cells. Proc Natl Acad Sci U A.

[10] Xiao R., Chen J.-Y., Liang Z., Luo D., Chen G., Lu Z.J., Chen Y., Zhou B., Li H., Du X. (2019). Pervasive Chromatin-RNA Binding Protein Interactions Enable RNA-Based Regulation of Transcription. Cell.

[11] Bampton A., Gittings L.M., Fratta P., Lashley T., Gatt A. (2020). The role of hnRNPs in frontotemporal dementia and amyotrophic lateral sclerosis. Acta Neuropathol..

[12] Gillentine M.A., Wang T., Hoekzema K., Rosenfeld J., Liu P., Guo H., Kim C.N., De Vries B.B.A., Vissers L.E.L.M., Nordenskjold M. (2021). Rare deleterious mutations of HNRNP genes result in shared neurodevelopmental disorders. Genome Med..

[13] Yates T.M., Vasudevan P.C., Chandler K.E., Donnelly D.E., Stark Z., Sadedin S., Willoughby J., Balasubramanian M., Broad Center for Mendelian Genomics, DDD study (2017). De novo mutations in HNRNPU result in a neurodevelopmental syndrome. Am. J. Med. Genet..

[14] Wang J., Choi J.-M., Holehouse A.S., Lee H.O., Zhang X., Jahnel M., Maharana S., Lemaitre R., Pozniakovsky A., Drechsel D. (2018). A Molecular Grammar Governing the Driving Forces for Phase Separation of Prion-like RNA Binding Proteins. Cell.

[15] Chi B., O’Connell J.D., Yamazaki T., Gangopadhyay J., Gygi S.P., Reed R. (2018). Interactome analyses revealed that the U1 snRNP machinery overlaps extensively with the RNAP II machinery and contains multiple ALS/SMA-causative proteins. Sci. Rep..

[16] Raczynska K.D., Ruepp M.D., Brzek A., Reber S., Romeo V., Rindlisbacher B., Heller M., Szweykowska-Kulinska Z., Jarmolowski A., Schümperli D. (2015). FUS/TLS contributes to replication-dependent histone gene expression by interaction with U7 snRNPs and histone-specific transcription factors. Nucleic Acids Res..

[17] Cooper-Knock J., Walsh M.J., Higginbottom A., Robin Highley J., Dickman M.J., Edbauer D., Ince P.G., Wharton S.B., Wilson S.A., Kirby J. (2014). Sequestration of multiple RNA recognition motif-containing proteins by C9orf72 repeat expansions. Brain.

[18] Cooper-Knock J., Robins H., Niedermoser I., Wyles M., Heath P.R., Higginbottom A., Walsh T., Kazoka M., Project MinE ALS Sequencing Consortium, Ince P.G. (2017). Targeted Genetic Screen in Amyotrophic Lateral Sclerosis Reveals Novel Genetic Variants with Synergistic Effect on Clinical Phenotype. Front. Mol. Neurosci..

[19] Gu Z., Churchman M., Roberts K., Li Y., Liu Y., Harvey R.C., McCastlain K., Reshmi S.C., Payne-Turner D., Iacobucci I. (2016). Genomic analyses identify recurrent MEF2D fusions in acute lymphoblastic leukaemia. Nat. Commun..

[20] Yasuda T., Tsuzuki S., Kawazu M., Hayakawa F., Kojima S., Ueno T., Imoto N., Kohsaka S., Kunita A., Doi K. (2016). Recurrent DUX4 fusions in B cell acute lymphoblastic leukemia of adolescents and young adults. Nat. Genet..

[21] Zhang M., Zhang H., Li Z., Bai L., Wang Q., Li J., Jiang M., Xue Q., Cheng N., Zhang W. (2022). Functional, structural, and molecular characterizations of the leukemogenic driver MEF2D-HNRNPUL1 fusion. Blood.

[22] Walker J.E., Saraste M., Runswick M.J., Gay N.J. (1982). Distantly related sequences in the alpha- and beta-subunits of ATP synthase, myosin, kinases and other ATP-requiring enzymes and a common nucleotide binding fold. EMBO J..

[23] Kzhyshkowska J., Schütt H., Liss M., Kremmer E., Stauber R., Wolf H., Dobner T. (2001). Heterogeneous nuclear ribonucleoprotein E1B-AP5 is methylated in its Arg-Gly-Gly (RGG) box and interacts with human arginine methyltransferase HRMT1L1. Biochem. J..

[24] Van Nostrand E.L., Freese P., Pratt G.A., Wang X., Wei X., Xiao R., Blue S.M., Chen J.-Y., Cody N.A.L., Dominguez D. (2020). A large-scale binding and functional map of human RNA-binding proteins. Nature.

[25] Erdős G., Pajkos M., Dosztányi Z. (2021). IUPred3: prediction of protein disorder enhanced with unambiguous experimental annotation and visualization of evolutionary conservation. Nucleic Acids Res..

[26] Kiledjian M., Dreyfuss G. (1992). Primary structure and binding activity of the hnRNP U protein: binding RNA through RGG box. EMBO J..

[27] Abramson J., Adler J., Dunger J., Evans R., Green T., Pritzel A., Ronneberger O., Willmore L., Ballard A.J., Bambrick J. (2024). Accurate structure prediction of biomolecular interactions with AlphaFold 3. Nature.

[28] Krissinel E., Henrick K. (2004). Secondary-structure matching (SSM), a new tool for fast protein structure alignment in three dimensions. Acta Crystallogr. D Biol. Crystallogr..

[29] Kelley L.A., Mezulis S., Yates C.M., Wass M.N., Sternberg M.J.E. (2015). The Phyre2 web portal for protein modeling, prediction and analysis. Nat. Protoc..

[30] Richardson C.C., Boyer P.D. (1981). The Enzymes.

[31] Garces F., Pearl L.H., Oliver A.W. (2011). The Structural Basis for Substrate Recognition by Mammalian Polynucleotide Kinase 3′ Phosphatase. Mol. Cell.

[32] Panhale A., Richter F.M., Ramírez F., Shvedunova M., Manke T., Mittler G., Akhtar A. (2019). CAPRI enables comparison of evolutionarily conserved RNA interacting regions. Nat. Commun..

[33] Castello A., Horos R., Strein C., Fischer B., Eichelbaum K., Steinmetz L.M., Krijgsveld J., Hentze M.W., Dassi E. (2016). Post-Transcriptional Gene Regulation Methods in Molecular Biology.

[34] Trendel J., Schwarzl T., Horos R., Prakash A., Bateman A., Hentze M.W., Krijgsveld J. (2019). The Human RNA-Binding Proteome and Its Dynamics during Translational Arrest. Cell.

[35] He C., Sidoli S., Warneford-Thomson R., Tatomer D.C., Wilusz J.E., Garcia B.A., Bonasio R. (2016). High-Resolution Mapping of RNA-Binding Regions in the Nuclear Proteome of Embryonic Stem Cells. Mol. Cell.

[36] Hein M.Y., Hubner N.C., Poser I., Cox J., Nagaraj N., Toyoda Y., Gak I.A., Weisswange I., Mansfeld J., Buchholz F. (2015). A human interactome in three quantitative dimensions organized by stoichiometries and abundances. Cell.

[37] Bernstein N.K., Hammel M., Mani R.S., Weinfeld M., Pelikan M., Tainer J.A., Glover J.N.M. (2009). Mechanism of DNA substrate recognition by the mammalian DNA repair enzyme, Polynucleotide Kinase. Nucleic Acids Res..

[38] Richter H., Katic I., Gut H., Großhans H. (2016). Structural basis and function of XRN2 binding by XTB domains. Nat. Struct. Mol. Biol..

[39] Jiao X., Chang J.H., Kilic T., Tong L., Kiledjian M. (2013). A Mammalian Pre-mRNA 5′ End Capping Quality Control Mechanism and an Unexpected Link of Capping to Pre-mRNA Processing. Mol. Cell.

[40] Dikfidan A., Loll B., Zeymer C., Magler I., Clausen T., Meinhart A. (2014). RNA Specificity and Regulation of Catalysis in the Eukaryotic Polynucleotide Kinase Clp1. Mol. Cell.

[41] Braglia P., Heindl K., Schleiffer A., Martinez J., Proudfoot N.J. (2010). Role of the RNA/DNA kinase Grc3 in transcription termination by RNA polymerase I. EMBO Rep..

[42] Heindl K., Martinez J. (2010). Nol9 is a novel polynucleotide 5′-kinase involved in ribosomal RNA processing. EMBO J..

[43] Golovanov A.P., Hautbergue G.M., Tintaru A.M., Lian L.Y., Wilson S.A. (2006). The solution structure of REF2-I reveals interdomain interactions and regions involved in binding mRNA export factors and RNA. RNA.

[44] Viphakone N., Hautbergue G.M., Walsh M., Chang C.T., Holland A., Folco E.G., Reed R., Wilson S.A. (2012). TREX exposes the RNA-binding domain of Nxf1 to enable mRNA export. Nat. Commun..

[45] van der Spek R.A.A., van Rheenen W., Pulit S.L., Kenna K.P., van den Berg L.H., Veldink J.H., Project MinE ALS Sequencing Consortium (2019). The project MinE databrowser: bringing large-scale whole-genome sequencing in ALS to researchers and the public. Amyotroph. Lateral Scler. Front. Degener..

[46] Karczewski K.J., Weisburd B., Thomas B., Solomonson M., Ruderfer D.M., Kavanagh D., Hamamsy T., Lek M., Samocha K.E., Cummings B.B. (2017). The ExAC browser: displaying reference data information from over 60 000 exomes. Nucleic Acids Res..

[47] Kircher M., Witten D.M., Jain P., O’Roak B.J., Cooper G.M., Shendure J. (2014). A general framework for estimating the relative pathogenicity of human genetic variants. Nat. Genet..

[48] Chen S., Francioli L.C., Goodrich J.K., Collins R.L., Kanai M., Wang Q., Alföldi J., Watts N.A., Vittal C., Gauthier L.D. (2024). A genomic mutational constraint map using variation in 76,156 human genomes. Nature.

[49] Havugimana P.C., Goel R.K., Phanse S., Youssef A., Padhorny D., Kotelnikov S., Kozakov D., Emili A. (2022). Scalable multiplex co-fractionation/mass spectrometry platform for accelerated protein interactome discovery. Nat. Commun..

[50] Cho N.H., Cheveralls K.C., Brunner A.-D., Kim K., Michaelis A.C., Raghavan P., Kobayashi H., Savy L., Li J.Y., Canaj H. (2022). OpenCell: Endogenous tagging for the cartography of human cellular organization. Science.

[51] Chi B., O’Connell J.D., Iocolano A.D., Coady J.A., Yu Y., Gangopadhyay J., Gygi S.P., Reed R. (2018). The neurodegenerative diseases ALS and SMA are linked at the molecular level via the ASC-1 complex. Nucleic Acids Res..

[52] Svetoni F., Frisone P., Paronetto M.P. (2016). Role of FET proteins in neurodegenerative disorders. RNA Biol..

[53] Nozawa R.-S., Boteva L., Soares D.C., Naughton C., Dun A.R., Buckle A., Ramsahoye B., Bruton P.C., Saleeb R.S., Arnedo M. (2017). SAF-A Regulates Interphase Chromosome Structure through Oligomerization with Chromatin-Associated RNAs. Cell.

[54] Oksanen M., Mastropasqua F., Mazan-Mamczarz K., Martindale J.L., Ye X., Arora A., Banskota N., Gorospe M., Tammimies K. (2026). Molecular interactome of HNRNPU reveals regulatory networks in neuronal differentiation and DNA methylation. Nucleic Acids Res..

[55] Noble C.G., Beuth B., Taylor I.A. (2007). Structure of a nucleotide-bound Clp1-Pcf11 polyadenylation factor. Nucleic Acids Res..

[56] Ghazy M.A., Gordon J.M., Lee S.D., Singh B.N., Bohm A., Hampsey M., Moore C. (2011). The interaction of Pcf11 and Clp1 is needed for mRNA 3’-end formation and is modulated by amino acids in the ATP-binding site. Nucleic Acids Res..

[57] Qamar S., Wang G., Randle S.J., Ruggeri F.S., Varela J.A., Lin J.Q., Phillips E.C., Miyashita A., Williams D., Ströhl F. (2018). FUS Phase Separation Is Modulated by a Molecular Chaperone and Methylation of Arginine Cation-π Interactions. Cell.

[58] West S., Gromak N., Proudfoot N.J. (2004). Human 5’ --> 3’ exonuclease Xrn2 promotes transcription termination at co-transcriptional cleavage sites. Nature.

[59] Shi Y., Di Giammartino D.C., Taylor D., Sarkeshik A., Rice W.J., Yates J.R., Frank J., Manley J.L. (2009). Molecular architecture of the human pre-mRNA 3’ processing complex. Mol. Cell.

[60] Martyr J.G., Zafferani M., Bailey M.A., Zorawski M.D., Montalvan N.I., Muralidharan D., Fitzgerald M.C., Hargrove A.E. (2025). Small molecules reveal differential shifts in stability and protein binding for G-quadruplex RNA. bioRxiv.

[61] Loughlin F.E., Lukavsky P.J., Kazeeva T., Reber S., Hock E.-M., Colombo M., Von Schroetter C., Pauli P., Cléry A., Mühlemann O. (2019). The Solution Structure of FUS Bound to RNA Reveals a Bipartite Mode of RNA Recognition with Both Sequence and Shape Specificity. Mol. Cell.

[62] Raguseo F., Wang Y., Li J., Petrić Howe M., Balendra R., Huyghebaert A., Vadukul D.M., Tanase D.A., Maher T.E., Malouf L. (2023). The ALS/FTD-related C9orf72 hexanucleotide repeat expansion forms RNA condensates through multimolecular G-quadruplexes. Nat. Commun..

[63] Lagier-Tourenne C., Polymenidou M., Cleveland D.W. (2010). TDP-43 and FUS/TLS: emerging roles in RNA processing and neurodegeneration. Hum. Mol. Genet..

[64] Giudice J., Jiang H. (2024). Splicing regulation through biomolecular condensates and membraneless organelles. Nat. Rev. Mol. Cell Biol..

[65] Meng E.C., Goddard T.D., Pettersen E.F., Couch G.S., Pearson Z.J., Morris J.H., Ferrin T.E. (2023). UCSF ChimeraX: Tools for structure building and analysis. Protein Sci..

[66] Schneider C.A., Rasband W.S., Eliceiri K.W. (2012). NIH Image to ImageJ: 25 years of image analysis. Nat. Methods.

