# Supplementary material for: hnRNPUL1 has a dead polynucleotide kinase domain that regulates RNA and protein interactions
Source: iScience. 2026 Mar 14;29(4):115360. doi: 10.1016/j.isci.2026.115360 (PMC13066744; doi:10.1016/j.isci.2026.115360)
Supplement: Document S1. Figures S1–S4 and Table S1 [file mmc1.pdf]

iScience, Volume *29*

Supplemental information

hnRNPUL1 has a dead polynucleotide kinase domain that regulates RNA and protein interactions

Carmen V. Apostol, Ang Li, Peter Daniels, Llywelyn Griffith, Johnathan Cooper-Knock, Elisa Aguilar-Martinez, Ivaylo D. Yonchev, Ashleigh G.R. Whelan, Pamela J. Shaw, Ian M. Sudbery, and Stuart A. Wilson

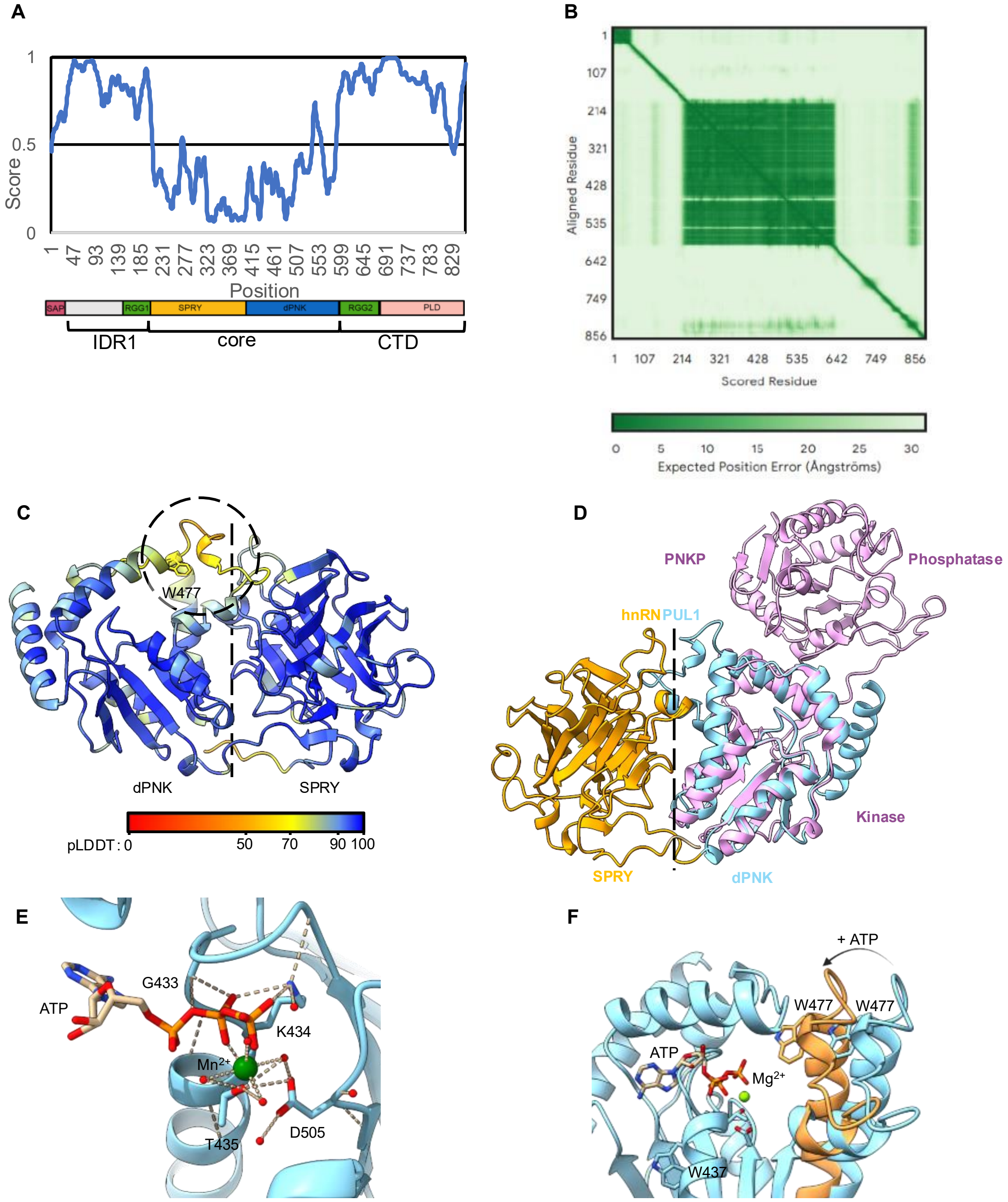

Figure S1. Structural analysis of hnRNPUL1, related to Figure 1. A) Per residue disorder prediction of hnRNPUL1, generated using IUPred3. Scores above 0.5 are expected to be disordered, while regions below 0.5 are predicted to be structured. B) Plot of Predicted Aligned Error for the Alphafold3 model of full-length hnRNPUL1. Dark green represents a small expected error in the distance between a pair of residues, while light green represents a large expected error. C) Per residue confidence scores (pLDDT) for Alphafold3 prediction of the central folded domain of hnRNPUL1. The sidechain of W477, located in a region predicted with low confidence relative to the rest of the core (encircled), is shown in stick form. D) The dPNK region of the central folded domain of hnRNPUL1 (415-600; light blue) superimposed onto PNKP (307-522; pink, PDB: 3ZVN) using the 'Matchmaker' function of ChimeraX, with an RMSD for aligned Cα pairs of 1.1 Å. E) Predicted hydrogen bond network involving T435 of the Walker A motif and D505 of the Walker B motif and metal coordination. The ATP, metal ion and water molecules used in the analysis come from the ligand-bound PNKP structure (3ZVN) superimposed onto

the hnRNPUL1 model. The GK residues also mutated in WA_m are highlighted. F) Model of potential conformational change within the hnRNPUL1 core upon ATP binding. The open and closed structures are mapped onto the apo and ligand-bound forms of PNKP, respectively (PDB:3ZVL and 3ZVN, DNA substrate omitted for clarity). The structural changes are mostly restricted to the W477-containing low confidence region from C.

A

RNA-Peptide crosslinking Studies:

	He et al 2016 RBR-ID	Castello et al 2016 RBDmap	Trendel et al 2019 XRNAX	Panhale et al 2019 CAPRI
hnRNPU	2-10, 13-21, 182-199, 232-244, 329-336, 466-486, 513-534, 542-548, 552-566, 614-650, 772-800	13-22, 256-265,424-433,495-510,566-572, 593-601, 627-635, 665-674, 797-814	621-635	247-265, 300-331, 352-384, 565-592, 697-825
hnRNPUL1		147-165, 691-721		146-223, 375-416, 461-498, 607-731
hnRNPUL2	27-58, 260-280, 296-313, 478-519, 557-569, 575-622, 670-682, 737-745	739-747		207-227, 593-601, 648-747

Key: He et al 2016[40] Castello et al 2016[38] Trendel et al 2019[39] Panhale et al 2019[37]

B

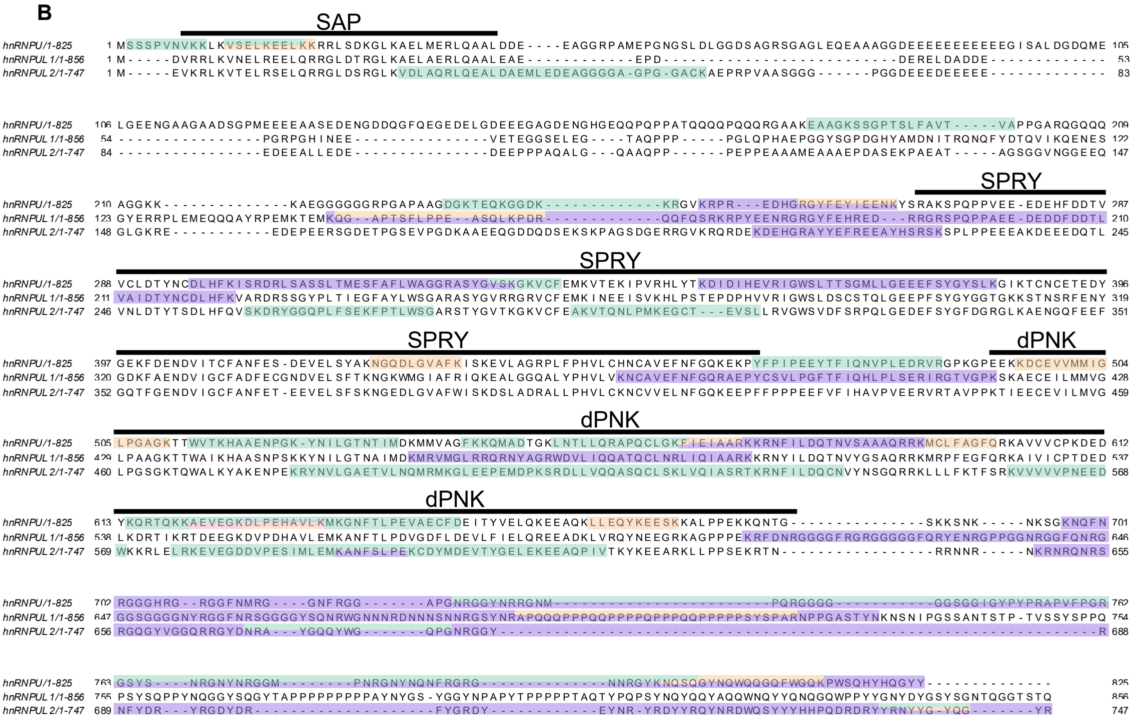

C

% Identity	hnRNPU	hnRNPUL 1	hnRNPUL 2
hnRNPU		49.66	50.42
hnRNPUL1	49.66		45.94
hnRNPUL2	50.42	45.94	

Figure S2. Conservation of RNA-peptide crosslinks in human hnRNPU family proteins, related to Figure 2.

A) List of observed RNA-protein crosslinked peptides retrieved from four mass spectrometry studies. B) RNA-protein crosslinked peptides mapped onto a Multiple Alignment using Fast Fourier Transform (MAFFT) of human hnRNPU family proteins. Where sequence positions are covered by multiple studies, colours are horizontally portioned. Putative globular domains are indicated by a black line, with boundaries defined as predicted for hnRNPUL1. C) Percentage identities between proteins aligned by MAFFT.

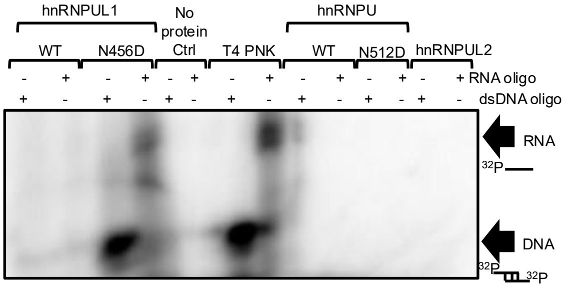

Figure S3. Polynucleotide kinase activity is not restorable with a point mutation in hnRNPU, nor hnRNPUL2, related to Figure 3.

Kinase assay using full-length hnRNPUL1, hnRNPU and hnRNPUL2 forms, ɣ-^{32}P ATP and RNA or dsDNA oligonucleotide substrates with free 5′ ends and 5′ overhangs. T4 PNK is used as positive control. N512D is the structurally equivalent mutation that restored catalytic activity to hnRNPUL1.

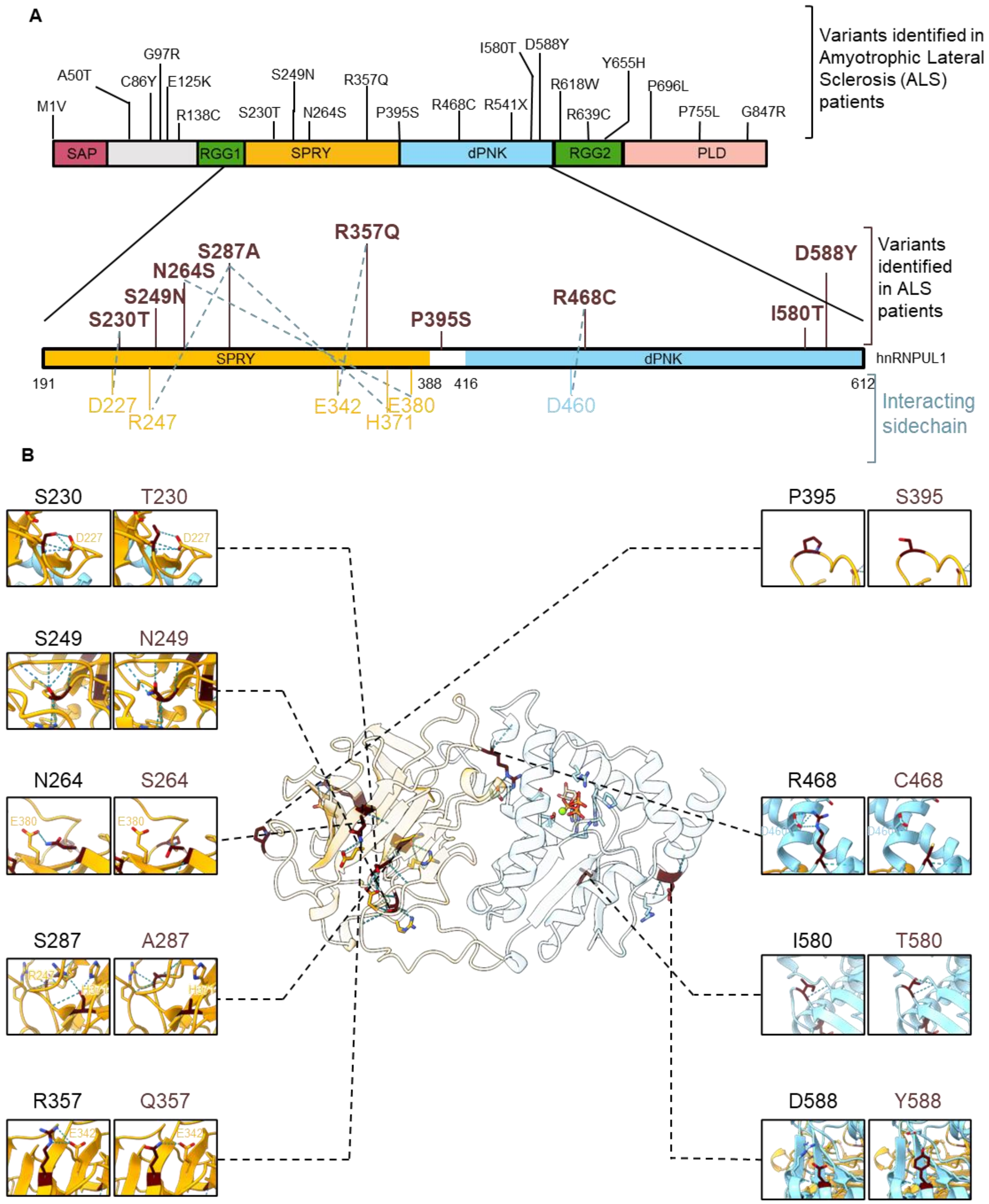

Figure S4. AlphaFold3 modelling of hnRNPUL1 variants in the SPRY-dPNK domain, related to Figure 4.

A) Schematic diagram of variants within the hnRNPUL1 SPRY-PNK domain (191-612) identified in ALS patients. AlphaFold3-predicted sidechain-sidechain interactions are represented by dotted blue lines in the lower panel. **B**) AlphaFold3-predicted fold disruptions in variants found in ALS patients. Residues found to be mutated are indicated in brown, boxed views represent aligned views of WT (left) or missense (right) hnRNPUL1 models. Models were generated in complex with an ATP molecule and Mg^{2+} ion, using the AlphaFold Server web portal.

Table S1. Rare variants in hnRNPUL1 found in ALS patients, related to Figure S4.

Mutation	Sex	Age of Onset (years)	Survival (months)	Phenotype	Familial/Sporadic	Dataset	CADD Score	gnomAD (v4.1.0) allele frequency	SNP ID / Genome variant (chr-pos-REF-ALT; GRCh38)
p.A50T	M	52	31	PMA	Sporadic	Project MinE	17.39	0.0001	rs755807621
p.S230T	M	69	17	ALS	Sporadic	Project MinE	22	6.196E-07	19-41276201-G-C
p.S249N	M	55	38	ALS	Sporadic	Project MinE	23.3	1.6x10-4	rs149059713
p.N264S	No information available	No information available	No information available	No information available	No information available	Project MinE	>20	3.3x10-5	rs750270062
p.G97R	No information available	No information available	No information available	No information available	No information available	Project MinE	22.8	Absent	19-41264792-G-C
p.C86Y	No information available - 2 patients	No information available	No information available	No information available	No information available	Project MinE	20.5	1.2x10-4	rs750372297
p.R138C	No information available	No information available	No information available	No information available	No information available	Project MinE	24.1	2.4x10-5	rs199709764
p.R357Q	No information available	No information available	No information available	No information available	No information available	Project MinE	>20	0.000003717	rs908333736
p.D588Y	No information available	No information available	No information available	No information available	No information available	Project MinE	>20	6.195E-07	19-41302739-G-T
p.P696L	No information available	No information available	No information available	No information available	No information available	Project MinE	20.4	8.3x10-6	rs764820631
p.P755L	No information available	No information available	No information available	No information available	No information available	Project MinE	32	1.6x10-5	rs752165954
p.G847R	No information available	No information available	No information available	No information available	No information available	Project MinE	>30	0.000005604	rs1454168002
p.M1V	No information available	No information available	No information available	No information available	Familial	UMass FALS	22.4	Absent	19-41264504-A-G
p.A50T	No information available	No information available	No information available	No information available	Familial	UMass FALS	20.9	0.0001	rs755807621
p.E125K	No information available	No information available	No information available	No information available	Familial	UMass FALS	23.5	1.7x10-4	rs144715337
p.S249N	No information available	No information available	No information available	No information available	Familial	UMass FALS	23.3	1.6x10-4	rs149059713
p.P395S	No information available	No information available	No information available	No information available	Familial	UMass FALS	23.7	0.000003717	19-41292428-C-T
p.R468C	No information available	No information available	No information available	No information available	Familial	UMass FALS	28.5	1.6x10-5	rs114983592
p.R618W	No information available	No information available	No information available	No information available	Familial	UMass FALS	22.2	1.6x10-5	rs755564298
p.R639C	No information available	No information available	No information available	No information available	Familial	UMass FALS	21.9	3.3x10-5	rs762181626
p.Y655H	No information available	No information available	No information available	No information available	Familial	UMass FALS	21.9	Absent	19-41302940-T-C
p.R541X	No information available	No information available	No information available	No information available	Familial	UMass FALS	39	Absent	19-41301638-C-T
p.I580T	No information available	No information available	No information available	No information available	Familial	UMass FALS	16.97	0.000006815	rs1304955393
